# Convergent Evolution of Mechanically Optimal Locomotion in Aquatic Invertebrates and Vertebrates

**DOI:** 10.1371/journal.pbio.1002123

**Published:** 2015-04-28

**Authors:** Rahul Bale, Izaak D. Neveln, Amneet Pal Singh Bhalla, Malcolm A. MacIver, Neelesh A. Patankar

**Affiliations:** 1 Department of Mechanical Engineering, Northwestern University, Evanston, Illinois, United States of America; 2 Department of Biomedical Engineering, Northwestern University, Evanston, Illinois, United States of America; 3 Department of Neurobiology, Northwestern University, Evanston, Illinois, United States of America; Lund University, SWEDEN

## Abstract

Examples of animals evolving similar traits despite the absence of that trait in the last common ancestor, such as the wing and camera-type lens eye in vertebrates and invertebrates, are called cases of convergent evolution. Instances of convergent evolution of locomotory patterns that quantitatively agree with the mechanically optimal solution are very rare. Here, we show that, with respect to a very diverse group of aquatic animals, a mechanically optimal method of swimming with elongated fins has evolved independently at least eight times in both vertebrate and invertebrate swimmers across three different phyla. Specifically, if we take the length of an undulation along an animal’s fin during swimming and divide it by the mean amplitude of undulations along the fin length, the result is consistently around twenty. We call this value the optimal specific wavelength (OSW). We show that the OSW maximizes the force generated by the body, which also maximizes swimming speed. We hypothesize a mechanical basis for this optimality and suggest reasons for its repeated emergence through evolution.

## Abstract 2

A mechanically optimal locomotory pattern of swimming has independently emerged eight times across three phyla, including vertebrates and invertebrates, demonstrating the role of mechanics in the convergent evolution of animals.

## Author Summary

How would animal life differ if it evolved again on Earth or any other habitable planet? If variation and selection can overwhelm all the other factors that might impede the approach to an optimum, then traits of animals that fulfill similar functional needs—such as camera-type eyes for seeing or wings for flying—are more likely to emerge independently and repeatedly. In aquatic animal swimming, one performance criterion is the Strouhal number (St), which specifies the frequency of fin movement for maximum propulsive efficiency in those animals that use the common “body/caudal fin” swimming mode, such as trout. Here, we use a combination of computational modeling, a robotic knifefish, and measurements of animal swimming behavior to study another widespread form of locomotion—“median/paired fin” swimming, used by animals as diverse as cuttlefish, triggerfish, and rays. Our studies provide quantitative evidence for a complementary performance criterion, called the optimal specific wavelength (OSW), which determines the wavelength of fin movement required for maximum propulsive force or thrust. Adherence to the OSW has independently emerged within eight clades of animals in three different phyla, including vertebrates and invertebrates, encompassing over a thousand species.

## Introduction

How would life look if it evolved again on Earth, or for that matter, on any other habitable planet? The question of the role of chance versus necessity in evolution is a foundational issue in biology [1–3]. Gould gave us the metaphor of the “tape of life” for the evolution of life and argued that if it were somehow rewound and started again, life would have taken a very different course [4]. Conway Morris has argued that, on the contrary, the laws of physics limit the number of good solutions that are within reach of evolution, and that therefore we should expect life to take a similar course upon rewinding [5]. Examples of convergent evolution, such as wings on insects, birds, and mammals, are considered supporting evidence for this hypothesis. But our understanding of convergent evolution, as reflecting the dominance of natural selection plus variation over factors such as developmental constraints, pleiotropy [6], phylogenetic inertia, genetic drift, and other stochastic processes [3], is held back by a lack of quantitative arguments. Such arguments would expose the links from physical principles to the biological phenomena and help us understand where evolution is likely to converge to the same result or diverge to a wide variety of solutions [7].

**Fig 1:**
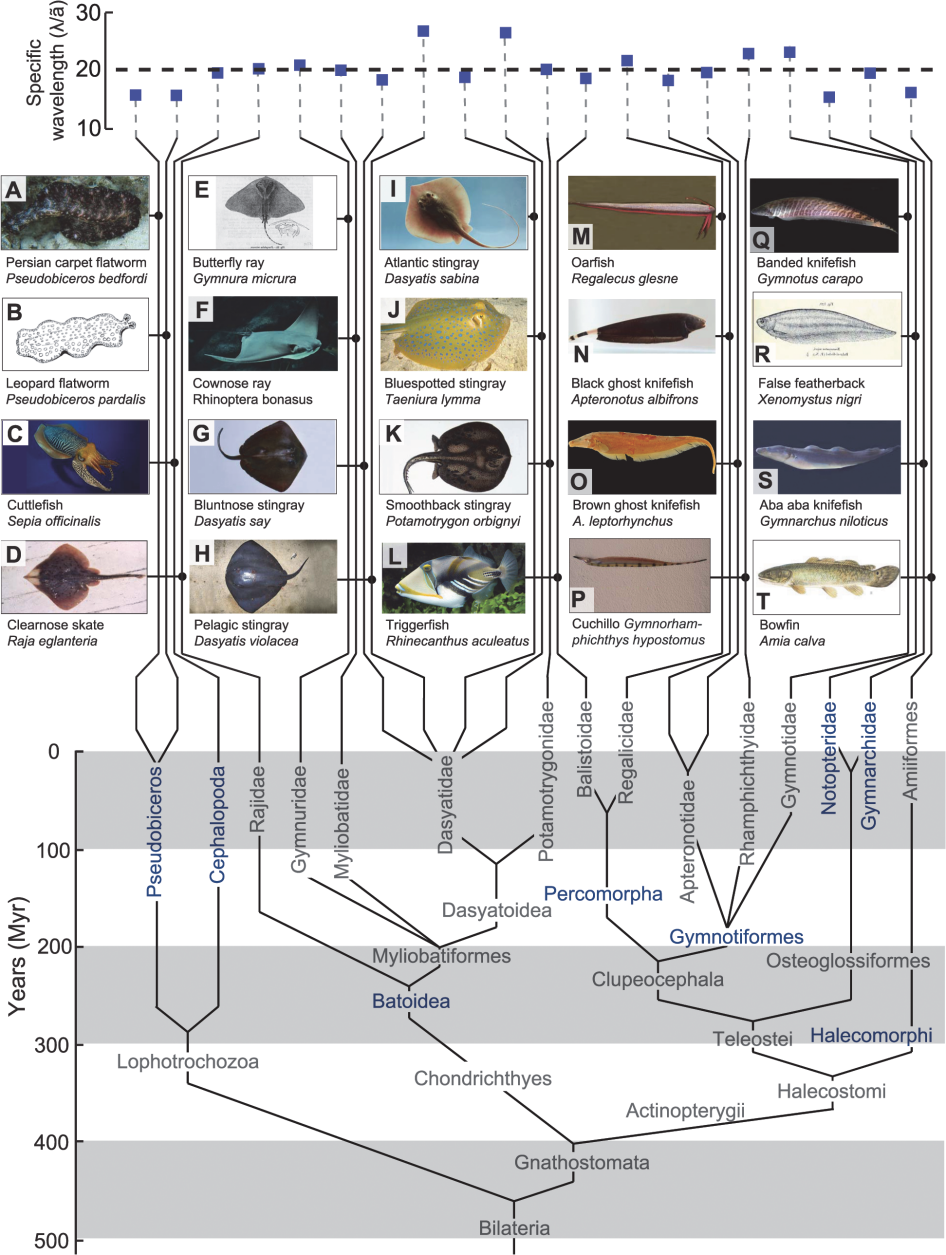
Undulatory median/paired fin swimmer phylogenetic relationships and SW = λ / *ã*, where λ and *ã* are wavelength and mean amplitude of undulations present along the fin, respectively. The eight instances of independent emergence of elongated median/paired fin swimming are highlighted in blue. The SW of these organisms and sources of the data are also tabulated in the S2 Table. Not shown here because of space constraints is the SW for the ray *Dasyatis americana*, which has an SW of 25.1, and the weakly electric knifefish *Eigenmannia virescens*, which use two counter-propagating waves on their fin during slow speed swimming and have an average SW of 17.7. See S4 Table. The following images are licensed under CC-BY: C) *Sepia officinalis* image courtesy of Hans Dappen. D) *Raja eglanteria* image courtesy of George Burgess. F) *Rhinoptera bonasus* image courtesy of Juan Aguere. J) *Taeniura lymma* image courtesy of Nicolai Johannesen. M) *Regalecus glesne* image courtesy of Sandstein. N) *Apteronotus albifrons* image courtesy of Clinton and Charles Robertson. O) *Apteronotus leptorhynchus* image courtesy of the Harvard Museum of Comparative Zoology. P) *Gymnorhamphichthys hypostomus* image courtesy of Mark Sabaj with support from IXingu Project (NSF DEB-1257813). S) *Gymnarchus niloticus* image courtesy of Masashi Kawasaki. All remaining images are public domain.

Here, we present just such arguments for a phenomenon that unifies a vast diversity of swimming organisms, from invertebrates, like cuttlefish, to vertebrates, like cartilaginous and bony fish. Unlike the case of the convergently evolved wing, a morphological feature, here the evolved feature is a pattern of movement [8–12] that occurs across a morphologically diverse set of moving appendages on aquatic animals. These animals swim by undulating elongated fins while keeping their body relatively rigid (Fig 1). Their fins run lengthwise with the body rather than crosswise to the body like the tailfin of a trout. The fins occur along the midline of the body, either along the belly or back (Fig 1N–1S, knifefish), as a dorsoventral pair (Fig 1L, triggerfish), or as a left–right pair (Fig 1C–1K, cuttlefish, skates, and rays). Movement by means of these fins is therefore called median/paired fin swimming, in contrast to the more common swimming style of fish like trout, in which a portion of the body and the caudal tailfin is moved, termed body/caudal fin swimming [13].

We find that in all cases where the wavelength of the traveling wave of median/paired fins has been documented during steady swimming, or can be extracted from readily available videos, the wavelength is about twenty times the mean amplitude (*ã*) of movement (mean = 19.5, standard deviation (STD) = 3.0, *n* = 22, Fig 1 and S2 Table). This includes cases, such as swimming of the cownose ray, in which the wavelength is greater than the fin length. These fins appear to only oscillate up and down to a casual observer but in fact undulate in the same manner as described earlier.

The ratio of the wavelength of an undulation to its mean amplitude is called the specific wavelength (SW). This parameter has been used in the past to study undulatory swimming [14–17] under water and under sand [15,16]. However, the finding in this work that there is a particular value of the SW that is observed across a diverse set of animals is new. We call this the optimal specific wavelength, or OSW. The OSW identifies the optimality in kinematics associated with undulatory patterns, in contrast to prior work [18], in which the focus was on quantifying bends solely due to propulsor flexibility.

Many of the organisms that swim with the OSW, including invertebrates such as cuttlefish and flatworms, jawed vertebrates such as rays and skates, and ray-finned bony fishes such as gymnotiformes, gymnarchids, and notopterids have no known ancestor that swam by means of median/paired fins (See S1 Methods Section 2). Swimming through undulation of median/paired fins with the OSW has therefore independently arisen at least eight times in three phyla (Chordata, Mollusca, and Platyhelminthes) among the Bilateria (Fig 1).

## Results

**Fig 2:**
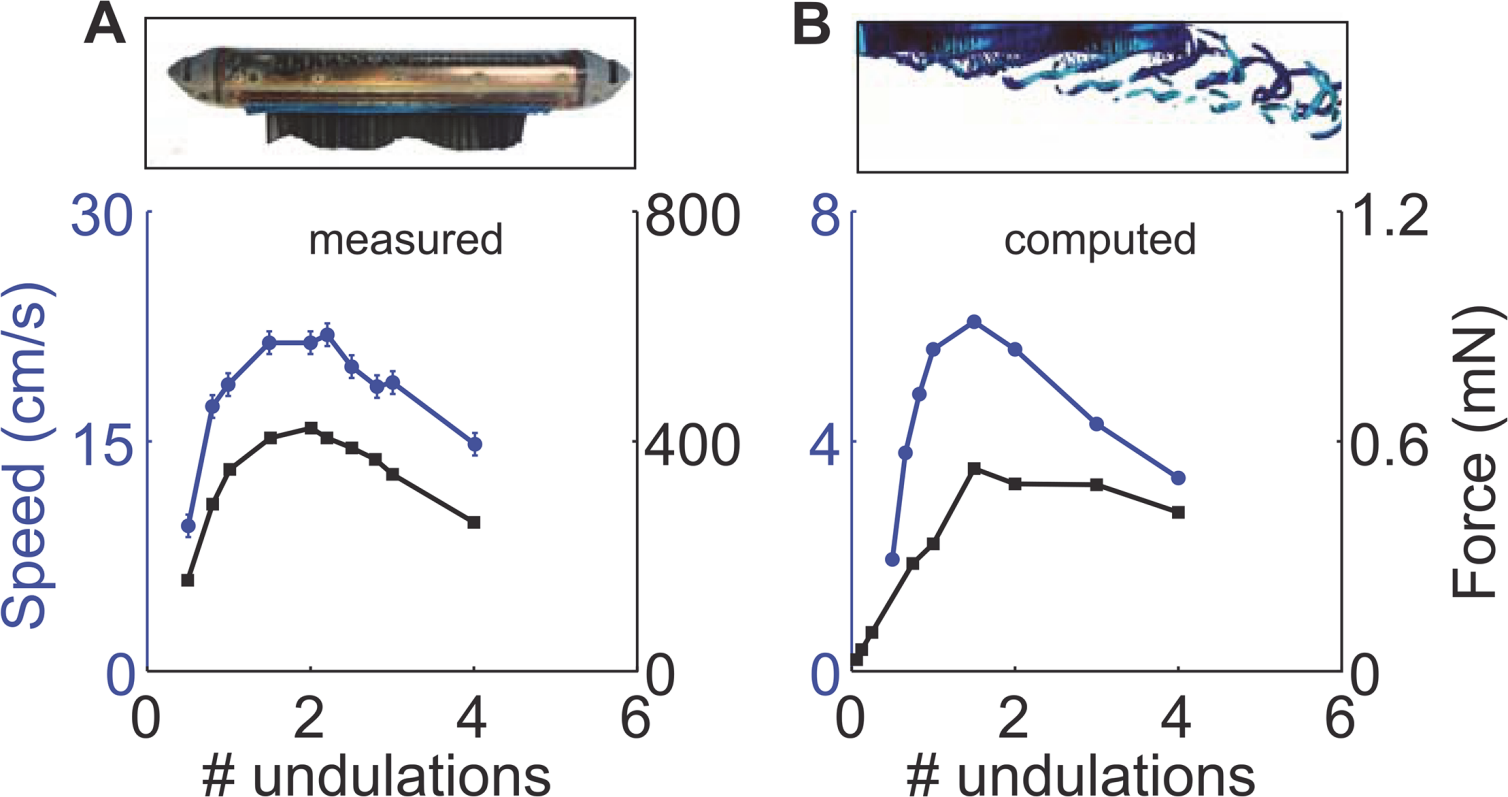
A) Experimentally measured axial swimming speed and propulsive force of a robotic knifefish as a function of the number of undulations along the fin (fin length divided by undulation wavelength). Modified from Fig 4B and Fig 5B of [20]. The error bars show STD from five trials of speed and force measurement experiments. The STD of error in measuring force is less than 1.5%, hence the error bars are barely visible at the scale of the plot. The fin was 32.60 cm long and 3.37 cm tall. For these experiments, *f* = 4 Hz and θ_max_ = 30°. Further details of measurements and error estimation can be found in our prior work [20]. Image of the robot taken by the authors. B) Axial swimming speed and propulsive force computed from simulations of a knifefish-sized fin (ten cm long, one cm tall, *f* = 2 Hz, θ_max_ = 30°) immersed in water, plotted against the number of undulations along the fin. In each plot, the left axis (in blue) and the blue curve corresponds to swimming speed, and the right axis (in black) and the black curve corresponds to propulsive force. Simulation code can be found at [26]. The data are available in S1 Data.

The values of SW, shown in Fig 1 for a variety of median/paired fin undulatory swimmers, converge to a narrow range (mean = 19.5, STD = 3.0, *n* = 22, S2 Table). Is there a mechanical optimum due to the physics of undulatory swimming that has driven evolution, in multiple instances, toward this narrow range of values of SW? An initial clue was obtained by our group from simulations of undulating neotropical knifefish fins [19] and measurements of the swimming speed of an undulatory fin robot [20]. Prior experimental work and current simulations show that swimming speed is highest when the number of undulations present along the fin (the fin length divided by the wavelength of the traveling wave progressing down the fin) is around two, similar to what is found in measurements of live knifefish [21,22]. The simulations were carried out on two sizes of fins. The first fin was similar in size and shape as the ventral elongated ribbon fin of a specific type of knifefish, the black ghost knifefish (*A*. *albifrons*, Fig 1N), where the fin measures 10 cm long by 1 cm tall. The second fin was a smaller fin measuring 2 cm long by 0.4 cm tall. A smaller fin was used because simulating many cases of a knifefish-sized fin for a more detailed parametric study, which we report later in the paper, was not practical due to limited computational resources. The experiments were with a robotic fin that was approximately three times larger in length and height (32.6 cm long by 3.6 cm tall) compared to the knifefish-sized fin. A larger robotic fin was built because of limitations of motor packing density for the smallest motors that could provide sufficient torque while providing an adequate number of artificial fin rays (32 rays [20], compared to 148 found in *A*. *albifrons* [23]). In Fig 2, we show the free swimming speed of the robot [20] and a simulated knifefish-sized fin [24,25] as a function of number of undulations along the fin. We also plot the propulsion force generated by undulating robotic and simulated fins that are prevented from translating along their long axis. We find that swimming speed and propulsive force are well correlated. This correlation is expected, since maximizing propulsive force or thrust is likely to enable the fastest swimming speed. Hereafter, we will present our analysis only in terms of propulsive force.

**Fig 3:**
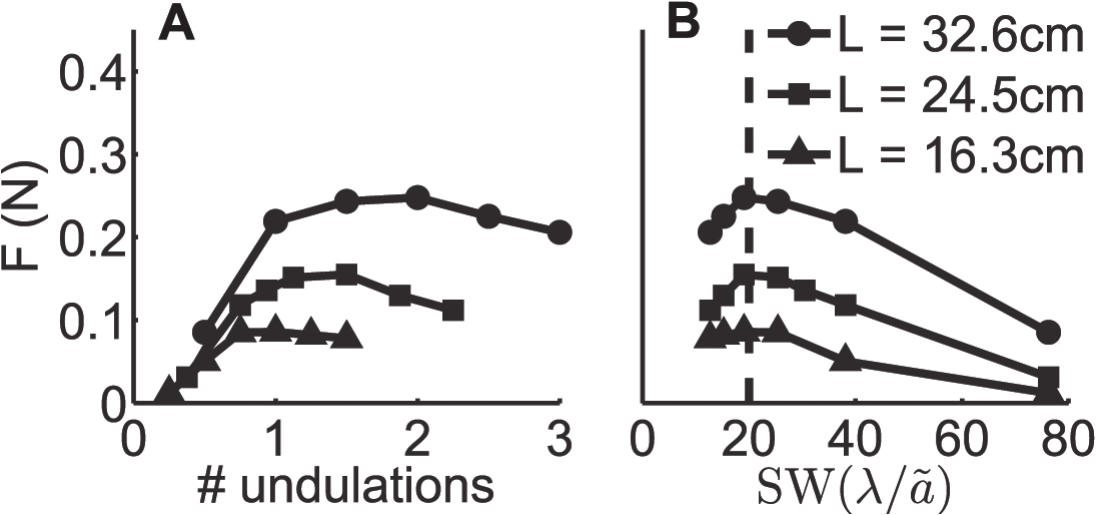
Effect of changes to fin length of a robotic knifefish on the optimal number of undulations and OSW. A) As the fin length is reduced from 32.6 cm to 16.3 cm, the optimal number of undulations reduces from 2 to 1. B) With the same changes in fin length, the OSW remains unaffected. Experimental parameters: θ_max_ = 20° (peak angle from midsagittal plane), fin height *h* = 5 cm, frequency of fin ray oscillation *f* = 3 Hz. The data are available in S2 Data.

As we varied the length of the simulated fin and the robotic fin for this study, while keeping other kinematic parameters the same, we were surprised to find that the number of undulations that resulted in maximal propulsive force (correlating to maximal free swimming speed) changed, as shown in Fig 3A. In this work, we show that if we examine force versus SW, instead of force versus number of undulations, the peaks of propulsive force all align at around a SW of twenty (Fig 3B). We find that the same is true when other kinematic and morphological parameters (amplitude of undulations, frequency, fin height, and fin shape) of the fin are varied (see below and S1 Methods Section 3). Because the propulsive force was maximal at an SW of around twenty across these examined parameters, we refer to SW ≈ 20 as the optimal SW or OSW.

### Robustness of OSW to Variations in Kinematics or Morphology

The axial propulsive force *F* depends on several physical variables of the problem:

F=fn(ρ,μ,f,λ,L,h,a), (1)

where *fn* denotes “function of," ρ is the density of water (0.9956×10^3^ kg/m^3^ at 25°C, varying less than 5% across the temperatures and salinities of aquatic organisms), μ is the viscosity of water (0.89×10^−3^ N ∙ s/m^2^ at 25°C), *f* is the frequency of oscillation of the fin, *λ* is the wavelength of undulation of the fin, *L* and *h* are the length and the height of the fin, respectively, *a* = *h* sinθ_max_ is the amplitude of undulation at the distal edge of the fin, and θ_max_ is the peak angle of excursion of the fin from the midsagittal plane (see S1 Fig). We assume a condition of steady temporally periodic flow in Equation 1. In our experiments and simulations, the force was generated by a undulating but non-translating fin. Hence, the fin velocity is not a physical variable of the problem. If the fin is attached to a swimming body then the swimming velocity is another dimensional variable of the problem. The steady swimming velocity also depends on the kinematic parameters listed in Equation 1.

Other physical variables of interest that depend on those listed in Equation 1 are noted next. *A* is the surface area of the fin, which is approximately given by *A* = *Lh*(1 + (*a* / λ)^2^). For the parameter space of interest in this work, *a* / *λ* ≪ 1, implying *A* ≈ *Lh*. A measure of the lateral velocity of oscillation of the fin is *V*_*l*_ = *f ã*, where *ã* is the average amplitude of oscillation of the fin. In the definition of SW, we use the average amplitude *ã* rather than the amplitude *a*. The reason is as follows: the propulsive force generated by an elongated fin depends on the amplitude of oscillation of the fin. For a given angular excursion, the amplitude of oscillation depends on the height of the fin, which typically varies along the length of the fin (see Fig 1, and S2 Fig for a schematic). The contribution to propulsive force from the part of the fin with greater height would be larger than that part of the fin which has relatively smaller height. Therefore, we use the average amplitude *ã* to define SW = λ / *ã* (and other parameters), where $\tilde{a}=h_{\text{mean}}\;\text{sin}(\theta_{\text{max}}^{\text{avg}})/2$. *h*_mean_ is the mean height of the fin and $\theta_{\text{max}}^{\text{avg}}$ is the mean angle of excursion of fin rays (for more details and a fully worked example, see S1 Methods Section 1 and S1 Table).

**Fig 4:**
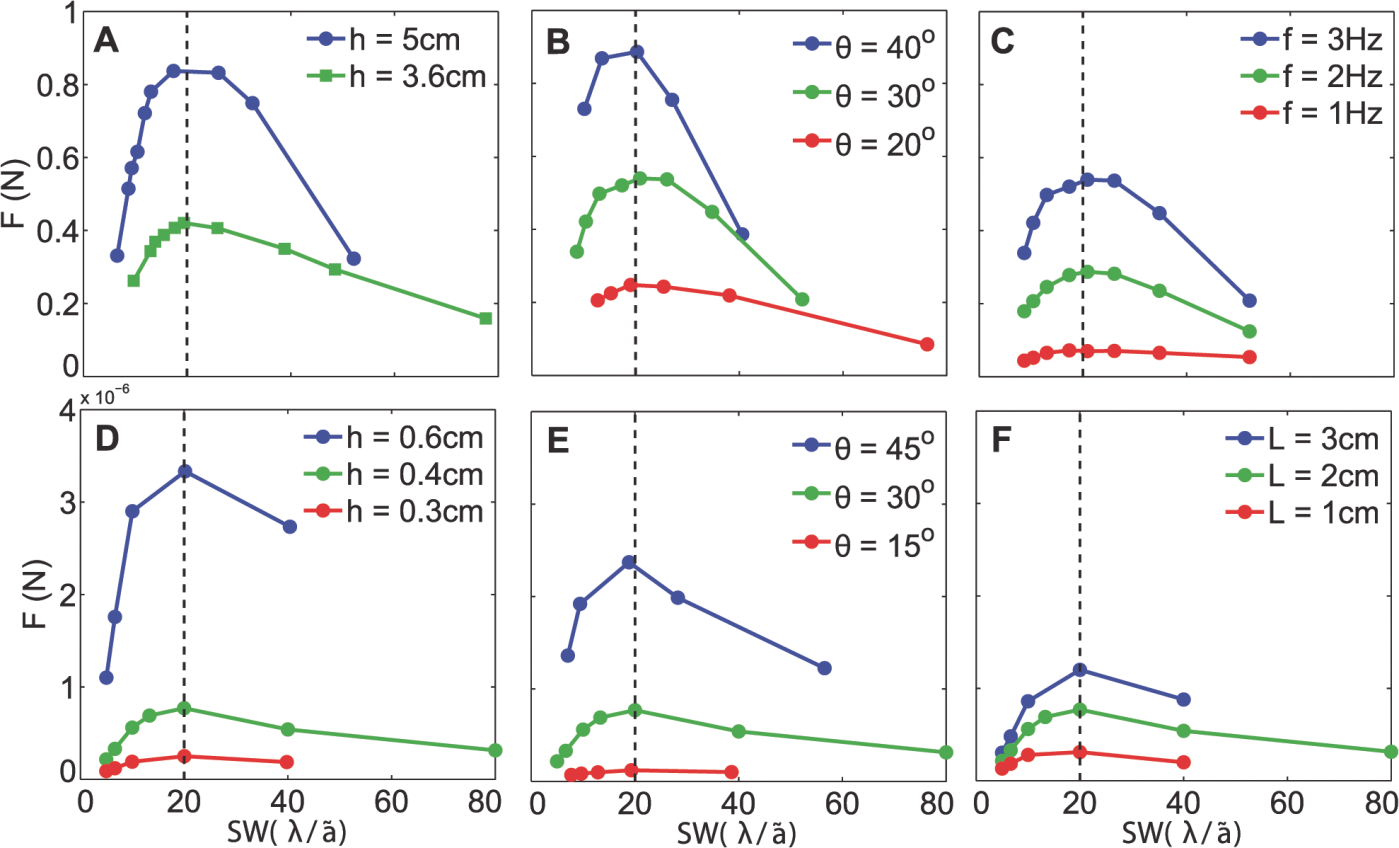
Results of a parametric study of the robotic fin plotted against SW in (A) to (C). (A) Fin height is varied, *L* = 32.6 cm, *f* = 4 Hz, and θ_max_ = 30°. (B) Angle of excursion is varied, *L* = 32.6 cm, *h* = 5 cm, and *f* = 3 Hz. (C) Frequency of undulations is varied, *L* = 32.6 cm, *h* = 5 cm, and θ_max_ = 30°. Results of a parametric study of the simulated fin plotted against SW in (D) to (F). (D) Fin height is varied, *L* = 2 cm, *f* = 1 Hz, and θ_max_ = 30° (E) Angle of excursion is varied, *L* = 2 cm, *h* = 0.4 cm, and *f* = 1 Hz. (F) Fin length is varied, *h* = 2 cm, *f* = 1 Hz, and θ_max_ = 30°. The fluid was water in all cases. The simulation code can be found at [26]. The data are available in S3 Data.

Equation 1 shows that there are eight physical variables including the propulsive force. The viscosity and density of water are known. Thus, the propulsive force *F* depends on the five remaining physical variables of which the wavelength, or equivalent SW, is one variable. We find that although *F* depends on multiple variables listed in Equation 1, the maximum is always at SW ≈ 20, irrespective of the other parameters. This is demonstrated in Fig 4. It is seen that the OSW did not change over the range of frequencies, angles of excursion, fin heights, and fin lengths shown. Further details of this parametric study are discussed in S1 Methods Section 3. In addition to the effect of rectangular fin height, the effect of different nonrectangular fin shapes on the OSW was also investigated. Fin shapes ranging from rounded convex to triangular (such as those used in a robotic cownose ray [27]) had no effect on the value of the OSW. This is discussed further in S1 Methods Section 4.

For the parameter space we examined, the qualitative trend of *F* with respect to SW is independent of the size of the fin. Consequently, the OSW is independent of size over the range examined here. Although our experiments and simulations are limited to fins between 2 cm and 32 cm in length, data from 22 species of swimming animals in Fig 1 show that the OSW holds over a wide range of lengths that span two orders of magnitude, from a 2 cm long *Pseudobiceros pardalis* to a 255 cm long *R*. *glesne* (see S2 Table). Beyond our sample of 22 species, there are over 1,000 median/paired fin species (either within the same genera or in higher taxonomic categories) that we predict will exhibit the OSW.

### Exclusion of Seahorse Data

We have chosen not to include the seahorse *Hippocampus hudsonius* within our results. This species is the sole exception we could find to the OSW, according to fin kinematics reported by Blake [28]. We decided not to include this case for the following reasons: the fin of the seahorse discussed in [28] was 2.1 cm long and around 1 cm deep. Blake reported that the fin moved with an amplitude of 0.63 cm at a frequency of 40 Hz while having 3.3 undulations. We cannot be confident that the reported kinematics are physically achievable by animals. To understand why, consider the kinematics of the black ghost knifefish [22]. The fin height is the same for the seahorse and the knifefish. The length of the seahorse fin is five times less than that of the knifefish. If the knifefish were to undulate its fin in an equivalent scenario, it would need to have around 15 undulations. The highest number of undulations along the fin of a knifefish is around four [22]. Thus, for conditions equivalent to the seahorse, the fin membrane would be subjected to extremely high strains and stresses, which seem physically unrealizable.

## Discussion

### Convergent Evolution and the OSW

Convergent evolution, the repeated emergence of a trait from an ancestor that did not have the trait, is the most compelling evidence for what has been called the “robust repeatability thesis” [3], the view that the evolution of animals can be explained by the comparative selective advantage of evolutionary survivors over their inferior counterparts. This is in contrast to Gould’s view—that quite different animal forms would emerge if evolution were to rerun its course. This view of macroevolution, the “radical contingency thesis,” [3] is that early patterns of animal evolution were generated by a selection process that was unrelated to the comparative fitnesses of the selected lineages.

What is the mechanism for macroevolutionary repeatability? In the language of the calculus of variations, these examples of convergent evolution—if correctly identified as such—imply that there is a gradient in the fitness landscape toward some optimum with respect to trait in question, and this gradient is large enough to overcome competing factors such as developmental constraints, pleiotropy [6], phylogenetic inertia, genetic drift, cases where optimality in one trait results in suboptimality in another trait (e.g., [29]), and approximations of the trait which provide local but not global optimality. With a sufficiently steep gradient in fitness in place and evolutionary dynamics capable of achieving near-optimal solutions, it is only a matter of time before the mechanism of selection with variation can arrive at the optimum. As the derivative of the trait with respect to fitness is stabilizing, departures from the optimum would be self-correcting over evolutionary time.

What is the evidence of a gradient in the case of the OSW? To address this question, we examine how variation in the SW affects force production (and therefore swimming speed) across the observed range of SW (Fig 1). We then compare the results to the variation in force production for SW outside of the observed range.

By using simulation and experimental results presented in S1 Methods Section 3, we found that for a SW between 15 and 25, corresponding to the observed natural range (Fig 1), the respective propulsive force decreases by around 7.5% at most from the optimal value (see S1 Methods Section 5 and S3 Table for details). Outside the naturally observed range of 15–25, the reduction in force grows significantly. When we consider an additional 25% deviation in SW, for a range of 10–30, the decrease in propulsive force is almost 25% (see S1 Methods Section 5 and S3 Table for details). The effect of any decline in propulsive force—even less than one percent—from what it is at the OSW is amplified over the vast number of undulations an animal may make in its life. Thus, we can only speculate that the 7.5% decline in force occurring over the observed variation in SW is not large enough to overcome the many causes of suboptimality listed above, whereas the 25% decline we find beyond this range is large enough to cause selection pressure toward the OSW. Additional research is needed to establish whether this hypothesis is true.

Finally, we note another aspect of the stability of the OSW, which is its insensitivity to changes in key kinematic variables such as frequency and amplitude, as well as to changes in fin geometry such as height and shape (Fig 4, S1 Methods Sections 3 and 4).

### Is the OSW an Optimal Regime for a Suboptimal Type of Swimming?

While the previous discussion shows that once an elongated fin species has arisen it may be costly to depart from the OSW, elongated fin swimmers constitute a minority amongst all swimmers. For example, there are approximately 1,000 median/paired fin swimming species within the jawed fishes (based on ascending to the nearest non-median/paired fin swimmer at each node of the tree presented in Fig 1) compared to 33,000 total fish species currently listed within FishBase. The fastest fish, and therefore the fish that are able to produce the highest amount of force, are body/caudal fin swimmers [30,31]. The question is therefore why the slower forms of swimming exhibited by median/paired fin animals would emerge and thrive despite the prevalence of body/caudal fin swimming in ancestral species.

A similar question has arisen in simulation studies that show that a light-sensitive patch of skin can evolve through several intermediate forms into an advanced camera-type lens eye in only a few hundred thousand years [32]—why, then, are there so many existing animals with intermediate forms of eyes? Nilsson and Pelger’s answer is that camera-type lens eyes are only the best solution for certain animal–ecosystem combinations [32]. Our answer is similar: body/caudal fin swimming makes little sense in isolation. It is only within particular ecological contexts that some types of animals are able to survive better with this type of swimming than with alternative approaches.

In particular, median/paired fin swimming appears to be a low speed, low cost of transport specialization [21,30,33]. The lower amplitudes of fin movement that are possible in median/paired fin swimmers, compared to the very high amplitudes possible when the high power axial musculature is used in body/caudal fin swimmers, is therefore an advantage instead of a liability due to the lower energetic cost of transport of median/paired fin swimming [30]. The fact that median/paired fin swimming is used at lower speeds should not be confused, however, with the concept of maximizing speed by swimming at the OSW. Even when swimming at lower speeds (or whatever speed for that matter, which is determined by frequency and amplitude), for a given set of parameters (amplitude of undulations, frequency, fin height, and fin shape), if an animal swims with elongated median/paired fins, then its speed can be maximized for that set of parameters by swimming at the OSW.

The ecological circumstances in which median/paired fin swimming will be favored over the typically faster, higher cost of transport body/caudal fin mode are not clear. If we consider one group specifically, such as the Gymnotiformes, a number of ecological features stand out as possible factors. First, they live in habitats of murky water and are active at night. Therefore vision—and high speed predation based on visual guidance—is not a factor. Second, periodic reductions in oxygen levels of the rivers they inhabit [33] may favor the lower cost of transport of median/paired fin swimming. Third, species within this group sense using electric fields, a mode of sensing where there is fourth-power attenuation of signal with distance [34]. This quartic attenuation implies a penalty on gross movement in sensory acquisition tasks, since typically sensing range is extremely short [34–36]. Therefore, precise, small, and slow movements are most effective. Finally, because the field generator is in the trunk, movement of the body causes large distortions of the emitted signal, possibly favoring holding the trunk rigid [37,38] rather than using it for propulsion. Given these constraints, the elongated fins that are universally present within the more than 150 species comprising Gymnotiformes may be favored, but clearly a tremendous amount of work would need to be done to assess the relative importance of all of these factors in giving rise to this one group of median/paired fin swimmers.

While the existence of body/caudal fin swimmers and the existence of median/paired fin swimmers may or may not be subject to robust repeatability, what is clear is that if median/paired fin swimming with elongated fins and semirigid trunks emerges—as it has independently on multiple occasions according to Fig 1—it is very probable that the specific trait of swimming at the OSW will also emerge. This is because of the thrust-maximizing property of the OSW, the existence of the gradient in thrust efficacy with a stabilizing derivative mentioned above, and the insensitivity of the OSW to variations in key fin movement parameters such as frequency and amplitude and to variations in fin height and shape.

Maximizing thrust ensures that speed can be maximized for a given set of parameters, irrespective of whether the animal swims energetically efficiently during cruising, or possibly less efficiently during escape or attack maneuvers. Thus, it seems likely that aquatic animals could benefit from adhering to the OSW at all—or at least at most—times. An example of using the OSW for thrust maximization even when swimming speed is not a concern is provided by the phenomenon of counterpropagating waves along a single fin. Gymnotid fish such as the black ghost knifefish *A*. *albifrons* [22,39] and the glass knifefish *E*. *virescens* will generate undulations from head to tail (called the head wave) and undulations from tail to head (called the tail wave) along its median anal fin, producing antagonistic forces [40]. We and collaborators have shown that this enhances stability and maneuverability [40]. Published videos show indications of this pattern being more widespread than gymnotid fish, such as the oarfish *R*. *glesne* [41] and cuttlefish [42]. For *E*. *virescens*, where counterpropagating waves have been analyzed more fully, while the angular amplitude is relatively constant between the two waves, there is a distinct difference in the wavelength. As the fin is deeper rostrally, for a similar angular excursion of each fin ray, the resulting mean lateral amplitude *ã* of the fin will be greater in the head wave than in the tail wave (for fin profile, see S2 Fig from [40]). The fish compensates for these differences in mean amplitude with a longer wavelength for the head wave than the tail wave, keeping the SW closer to 20 than if a constant wavelength were present throughout the fin (see S4 Table). *A*. *albifrons* follows a similar trend, though more data is needed for a full analysis. Maintaining OSW for the head and tail counterpropagating waves maximizes the counteracting thrust forces which lead to increased stability and maneuverability [40].

Despite these uses of the OSW for force maximization, it should be noted that median/paired fin fish are able to switch to the higher force production mode enabled by body/caudal fin swimming when absolutely needed, such as C-starts in knifefish [43], gait changes in median/paired fin swimmers to body/caudal fin swimming as speed increases [30], and oarfish transitioning to anguilliform swimming for higher speed (for reference see the videos from [41]). A natural question is whether body/caudal fin swimmers, or those that undulate their entire body, such as eels and lamprey—or even terrestrial animals like snakes—abide by an OSW of ≈ 20. It is likely that some related criteria exist but the optimal value of the SW may be different, or it may not be a constant as in case of the animals considered here that swim by means of elongated fins while keeping their trunk semirigid. In this context, we note that in the characterization of sandfish swimming (effectively, a body/caudal fin swimmer in sand), Maladen et al. [14,15] find that the swimming speed of the sandfish is optimal at SW = 5. The issue of the applicability of an OSW-like optimum to other modes of swimming, although not within the scope of this work, merits future investigation.

### How the OSW Is Implemented

One obvious way in which swimming animals could adhere to the OSW is through neural control. Whether at the level of spinal circuitry, or through descending control, this control would enforce the OSW through changing amplitude and wavelength so as to maintain a fixed ratio of around 20, as discussed below in remarks discussing the implications of the OSW for controlling robots. While outside the scope of this study, this hypothesis warrants further investigation, perhaps using the weakly electric fish model system, which is heavily used within certain domains of neuroscience [44].

Another approach is to build the OSW into the material properties of the structures that comprise the fin. The fin is made of a collagenous membrane that connects adjacent fin rays in teleosts [45] and a muscle and collagen matrix in cuttlefish [46,47]. This connective tissue limits the maximal strain, and thus the curvature of the fin cannot increase beyond a certain value. Suppose the amplitude of movement is increased for a given wavelength. This would increase the curvature and strain in some parts of the membrane. If the maximal allowable strain is being approached, then the material would relax to a larger wavelength for the new larger amplitude. A demonstration of this principle is seen in experiments in which the anterior end of a long, flexible, free-swimming foil is oscillated [48]. When the amplitude of a traveling wave along the foil increased by slightly over a factor of two, the wavelength increased by a factor of 1.6 (see Fig 4, [48]). Interestingly, the primary structural protein of aquatic animal fins, collagen, is an ancient protein predating the divergence of the three phyla where we have observed the OSW [49].

Whether by neural control or by material properties, there is an additional aspect of swimming by median/paired fins that could facilitate genetic aspects of the implementation of the OSW. For the more prevalent body/caudal fin swimming style, there are a host of other functions served by the body besides propulsion, such as holding internal organs, or providing a mirror-like surface to facilitate camouflage [50,51]. Having to move the mass of the trunk for propulsion, including such things as internal organs, is a disadvantage for the body/caudal fin style of swimming.

In contrast to most body/caudal fin swimmers, median/paired fin swimmers use their fins purely as thrust elements. In moving fluid to generate thrust, they are mostly putting energy into moving fluid instead of the mass of the membranous fin. Therefore, the collection of genes that give rise to axial elongation [52,53] and the associated elongated fins used for thrust production may have few other functions, perhaps facilitating their approach to an optimum in comparison to the genes controlling body shape in swimmers that move their trunk for swimming [6]. Further evidence in support of this idea is the finding of convergently evolved anatomical specializations for one type of body/caudal fin swimmer that minimizes trunk movement during tail fin movement, resulting in a similarly pure thrust element [12]. These are the thunniform swimmers such as tuna and lamnid sharks which largely restrict movement to their caudal fin propulsor, and in doing so are able to swim faster than most other fish. We hypothesize that regulation of the OSW, axial elongation, and fin elongation in the phyla in which we observe these traits may reflect the action of common genetic networks, as has been shown to underlie convergent evolution in the case of the electric organ [54].

### Predicting the number of fin undulations

Based on the OSW and the measured mean undulation amplitude *ã*, the number of undulations that should be present along the fin of a swimming animal, cruising at any velocity, can be predicted. This is given by

$${\text{NU}}_{\text{predict}}=\frac{L}{\text{OSW}\;\tilde{a}}.$$ (2)

**Fig 5:**
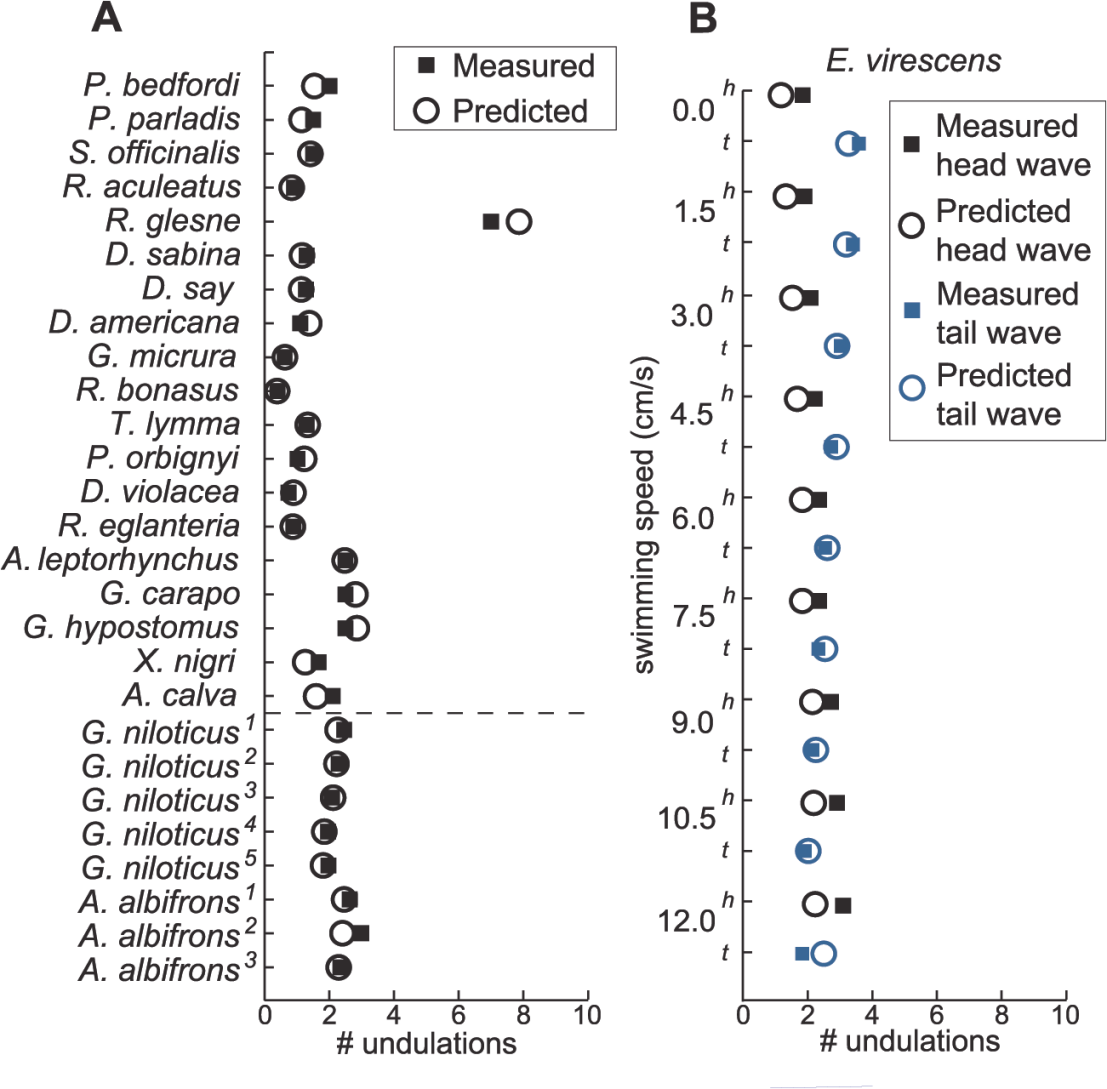
Predicted number of undulations along a fin (open circles) compared to measured number of undulations (filled squares) for the 20 species shown in Fig 1, plus *D*. *americana* in (A) and *E*. *virescens* in (B) (same data sources as listed in Fig 1). The order on the left plot is the same as Fig 1, except we have moved *G*. *niloticus* and *A*. *albifrons* to below the dashed line. Below the dashed line, the superscripts indicate data at different swimming speeds: For *G*. *niloticus*, 1 = 19.7 cm/s, 2 = 21.2 cm/s, 3 = 22.3 cm/s, 4 = 23.8 cm/s, 5 = 26.6 cm/s. For *A*. *albifrons*, 1 = 14.9 cm/s, 2 = 19.7 cm/s, and 3 = 32.6 cm/s. When the swimming velocity increases, amplitude is increased and the number of undulations is decreased to maintain an SW that is close to the OSW. (B) compares the predicted and measured number of undulations of both the head and the tail waves of the counterpropagating undulations in *E*. *virescens* averaged over five fish. The trends seen are largely due to the change in the lengths of the head and tail waves as the point where the two waves meet shift caudally with increased forward swimming speed. The STD across these data ranges from 0.15 to 0.67 and is not shown for clarity. The data are available in S4 Data.

We plot the measured number of undulations along with the predicted number of undulations in Fig 5 for all the same animals shown in Fig 1, using measurements of mean amplitude and assuming an OSW = 20. The number of undulations predicted is in excellent agreement with the observed number.

These predictions are for a single swimming velocity for each species. However, in two cases we have fin undulation data across several swimming velocities. Here too, the fin follows the pattern dictated by the OSW. The data for the African aba aba knifefish (*G*. *niloticus*) and South American black ghost knifefish (*A*. *albifrons*) below the dashed line in Fig 5A are at different swimming speeds. The superscripts on *G*. *niloticus* and *A*. *albifrons* relate to swimming speed, with the superscript number ^1^ corresponding to the slowest speed and higher numbers to higher speeds. The data show that as these fish swim faster, they reduce the number of undulations along their fins. This reduction in undulations along the fin is most likely because this is necessary to continue swimming at the OSW. To swim faster, the knifefish have to either increase the amplitude of fin undulations, or their frequency, or both. An important question for future investigation is whether the motor systems of undulatory animals provides for independent control of key kinematic parameters such as amplitude and frequency. For example, in a biologically inspired model of how coupled oscillators generate traveling waves along the anal fin of knifefish, the drive to the initial oscillator controlled frequency, while the gradient in drive across oscillators controlled the number of undulations [22], a situation in which there is no independent control of these key kinematic parameters. For the data plotted in Fig 5, to swim faster, the knifefish increase both frequency and amplitude [22,55]. Consequently, to compensate for the increase in amplitude, the wavelength is decreased to maintain the OSW.

Because the SW is independent of frequency, if an animal is swimming at the OSW and only frequency is varied to change speed, then the organism does not have to change the number of undulations along the fin to maintain the OSW. It appears that frequency control dominates at lower speeds for knifefish [21,22], which likely underlies the observation of a relatively constant number of undulations along the fin as swimming speed changes in a variety of electric and nonelectric knifefish [21,22]. Rosenberger [56,57] also mentions how some species of rays such as *Gymnura micrura* and *T*. *lymma* exhibit changes in fin motion with swim speed. As *G*. *micrura* and *T*. *lymma* increase their swimming speed, the number of undulations goes down. Rosenberger [57] also reported that *T*. *lymma* used smaller amplitudes at lower speeds. Consequently, to maintain the OSW, it would have to use more undulations when swimming slowly compared to when it is swimming rapidly using larger amplitudes. The same change in amplitude with speed has been mentioned qualitatively for *G*. *micrura* [56], and we would predict that quantitative measures would show that the OSW-prescribed pattern holds for this animal as well during changes in speed.

A final example of the correlation between amplitude and number of undulations is provided by the phenomenon of counterpropagating waves along a single fin discussed above. Despite traveling waves passing along the fin in opposite directions along fin membrane of differing depth (resulting in different amplitudes for similar angular excursions across fin rays), fish adjust the wavelength of undulations so as to maintain the OSW, as shown by Fig 5B.

### The OSW and St

A nondimensional number commonly used to characterize the swimming of body/caudal fin swimmers is the St (St = 2*fa* / *U*, where *U* is the swimming speed, 2*a* is the maximum peak to peak amplitude of the lateral fin excursion, and *f* is the frequency of tail beating). It has been reported that body/caudal fin swimmers cruise at an St between 0.2–0.4 [8,58]. It has also been reported, based on a meta-analysis of steady undulatory swimming, that the St varies over a much larger range of 0.2–1.8 [59]. Prior studies suggest that the basis of St between 0.2–0.4 in animals is high propulsive efficiency, which may relate to maintenance of attached flow [8] and hydrodynamic resonance [60], among other factors. Eloy [61], using Lighthill’s elongated body theory [62], has shown that an St between 0.15 and 0.8 minimizes energy expenditure and maximizes Froude efficiency. In swimming live knifefish, the St ranges between 0.3 and 0.6 (from data in [22]), the same range found in a study reporting data from a robotic knifefish as well as computational fluid simulations of knifefish fins [63].

A quantitative relationship between the St and the SW follows from their definitions:

$$\text{St}\;\eta_{wave}\;\text{SW}=\gamma ,$$ (3)

where $\eta_{\text{wave}}=\frac{U}{V_{w}}$ is the wave efficiency, which is the ratio of the velocity of a swimmer to the velocity of the traveling wave (frequency × wavelength) along the fin (see [64], where 1–*η*_wave_ is the “slip”). The wave efficiency can, in theory, take any value between zero and one. It has been reported that for high Froude efficiency, the wave efficiency should be in the range of 0.4–0.8 [61]. The wave efficiencies observed in swimming animals ranges from 0.1 to 0.9 [59]. The factor *γ* = 2*a* / *ã*, where 2*a* is the maximum peak to peak amplitude, while *ã* is the mean amplitude as previously defined, is due to the difference in how the amplitude parameter is defined in the formula for St and how we define it for the SW. *γ* ranges from four, for a rectangular fin similar to those of knifefish, to around eight for a deep triangular fin similar to those of some batoid fishes.

**Fig 6:**
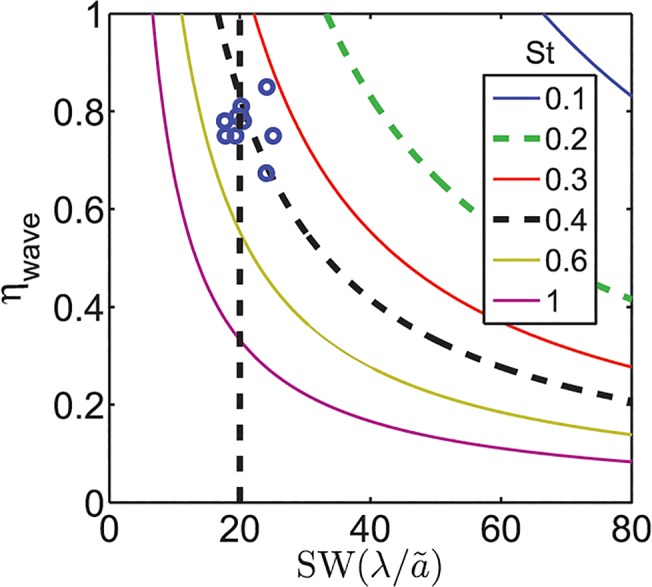
Isolines of St against SW on the *x*-axis and wave efficiency on the *y*-axis. St was computed using Equation 3, in which the mean value of *γ* = 6.7 for batoid fish, presented in Fig 1, was used. The OSW is highlighted by a dashed vertical line. The dashed contour lines correspond to the St range of 0.2–0.4. Also plotted are the data points (open circles) of the batoid fishes. The data are available in S5 Data.

Given St and SW, the wave efficiency, *η*_wave_, is determined according to Equation 3 for a given *γ*. For the range of *γ* from 4–8, a SW of 20 combined with the reported range of St of 0.2–0.4 [8,58] results in high wave efficiencies. As an example, the relationship from Equation 3 for *γ* = 6.7, that is typical of batoid fish, is shown graphically in Fig 6. The wave efficiencies are all above 0.6. Therefore, while St and SW are complementary, the observed values are also consistent with each other.

Although the St and the SW are related according to Equation 3, these two parameters are not equivalent. To appreciate this, three considerations should be noted. The first is that St and SW quantify different physical parameters of the problem. The St number quantifies information about the amplitude and frequency of undulations relative to the swimming speed, while the SW number quantifies the wavelength of undulations relative to amplitude. The difference between these two parameters is highlighted by situations in which median/paired fin swimmers undulate their fins while their swimming speed is zero to enhance stability and maneuverability [40], the phenomenon of counterpropagating waves mentioned above. In this situation, St approaches infinity, while the fish maintains the optimal SW.

The second consideration is that the optimal value of St (0.2–0.4) recommends the frequency that maximizes the propulsive efficiency (the ratio of thrust power to the power spent by the fin) of the fin [58] without any recommendation for the wavelength of undulations. A high propulsive efficiency case could be possible with large thrust or small thrust or other values. Of all the cases that have comparable propulsive efficiency, the case with maximum thrust is desirable since it will also lead to a fast swimming speed. For aquatic animals swimming by means of elongated median/paired fins, the OSW recommends an appropriate wavelength so that the propulsive thrust is also maximized. Thus, the St and the OSW are complementary and can help identify two physical parameters of the problem—frequency and wavelength—such that a median/paired fin animal swims with high propulsive efficiency and high thrust.

Finally, the third difference between the St and the OSW arises from consideration of how these two parameters can enter into the control of advanced underwater robots that use undulating fins. The St incorporates swimming velocity, the time derivative of position, which—like orientation—is termed a group variable in mechanics. The SW only refers to variables internal to the body (amplitude and wavelength), frequently called shape variables. Often in mechanical systems there exists full control authority over shape variables, meaning any time evolution of the variables can be specified to arbitrary precision. The group variables then evolve through nonlinear dynamics involving the shape variables [65]. In the case of swimming velocity, for example, these dynamics are governed by the Navier-Stokes equation at intermediate Reynolds numbers. Control of robotic undulators for advanced underwater robots in the future may involve the relatively simple control of proportionately changing wavelength with amplitude for maximizing force production by maintaining the OSW. However, maintaining the St within a specific range would require estimating swimming velocity with a sensor or forward model, accompanied by control which accounts for the nonlinear effects that the shape variables such as frequency and amplitude have on the velocity. To the extent that the neural or mechanical control of a fin in an aquatic animal proceeds along similar lines, it may also be the case that adherence to the OSW is a relatively direct function of variables under the control of the animal.

### A Hypothesis for Competing Mechanisms Underlying the OSW

**Fig 7:**
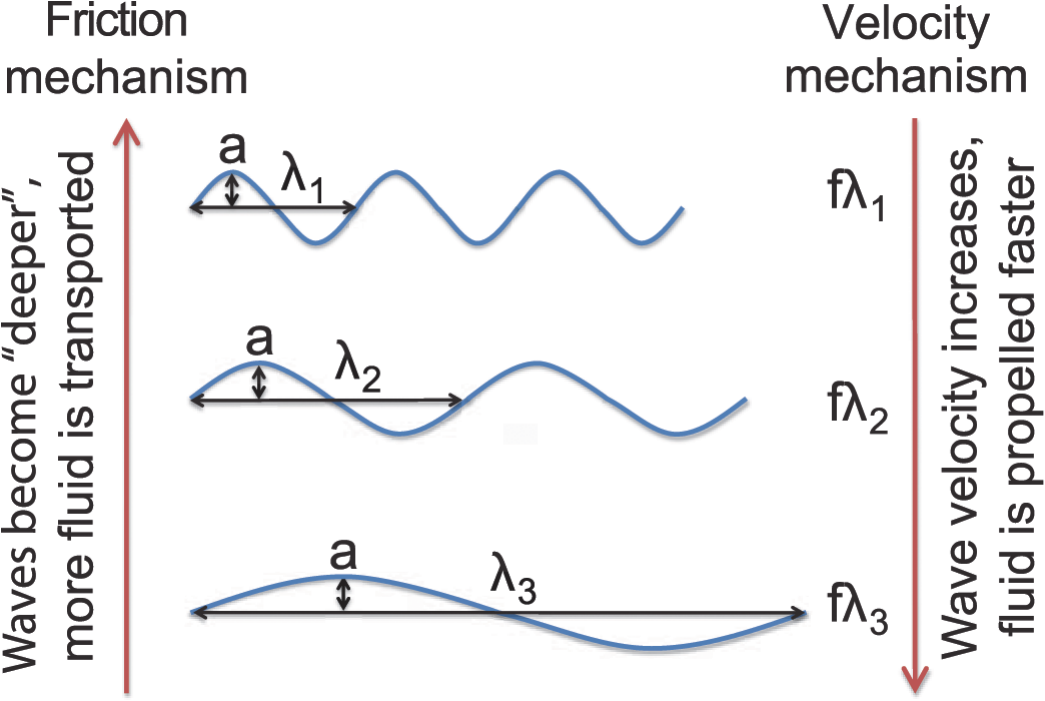
A schematic of a hypothesis for two competing mechanisms that could lead to the optimal propulsive force and therefore swimming speed. Keeping the amplitude, length, and frequency fixed, as the wavelength is increased (top to bottom), the wave velocity increases proportionally (indicated by the downward arrow on the right). As the wave velocity increases, the fluid is propelled backward at higher velocity. At the same time, the effectiveness with which the fin can trap and transport the fluid decreases (indicated by the upward arrow on the left).

What is the physical basis of the optimal value of the SW? The SW can be related to the peak steepness (slope) of the surface of an undulatory fin. The value of the peak slope corresponding to the OSW will depend on the fin geometry and the shape of the undulatory wave. For a 2D sinusoidal wave at the OSW, the angle made by the fin surface at the location of the peak slope is 17.44°. Our results indicate that for a given amplitude, the propulsive force is maximum at an intermediate peak steepness of the surface of the fin: very steep and very shallow are both suboptimal. We hypothesize that the optimal peak steepness may be rationalized in terms of two competing mechanisms (Fig 7). A steeper surface (smaller SW) of the fin should be able to trap and transport the fluid backward more efficiently. The increase in backward fluid momentum should lead to a stronger propulsive force at small SW. We call this the “friction mechanism" in Fig 7. A shallower surface (larger SW) of the fin leads to larger wave velocity resulting in the fluid being propelled backward at higher speed. Faster fluid velocity should increase backward fluid momentum and should lead to a stronger propulsive force at large SW. We call this the “velocity mechanism” in Fig 7. Thus, we speculate that SW influences two separate physical mechanisms with opposite trends. These competing mechanisms would lead to an optimal value of propulsive force at an intermediate value of SW.

## Methods

### Experimental Setup

The experiments were carried out using an updated version of the “Ghostbot” (S4 Fig, panel A) used in a number of previous studies [20,39,66]. The Ghostbot generated propulsive force using an elongated fin inspired by the ventral fin of a knifefish. The fin was 32.6 cm long and 5 cm deep. The fin was composed of 32 discrete fin rays which were driven by individual motors housed in a cylindrical body. The rays were connected by a Lycra fabric which constitutes the fin membrane. A more detailed description of the Ghostbot can be found elsewhere [20].

### Experimental Procedure

The axial force generated by the ribbon fin was measured similar to the methods presented in our earlier work [20]. The robot was submerged into a custom built flow tunnel and was suspended from above on a frictionless air bearing system allowing near-frictionless motion (S4 Fig, panel B). The dimensions of the working area of the tunnel were 33 cm wide, 32 cm deep, and 100 cm long. The robot was oriented horizontally and fixed in place in the water. A calibrated single axis force transducer (LSB200, Futek, Irvine, CA, USA) was placed between the air-bearing platform and mechanical ground in the axial direction of the robot so that longitudinal forces could be measured. For each trial, voltages from the force transducer were recorded at 1,000 Hz over a period of approximately 20 seconds while the fin was undulating in stationary water. The last 8 s of data were averaged. A zero measurement was also taken while the robot’s fin was at rest (not undulating) and was subtracted from the average voltage measurement from each trial. Voltage data were then converted into force units based on the calibration, which had a maximum nonlinear error of 0.034%. Details of the experiment in which free swimming velocity of the robot was measured can be found in prior work [20].

### Error Measurements

The error bars in Fig 2 show STD of error from measurements of speed and force detailed in the following. The swimming speed of the robot was measured in five consecutive trials with the same kinematic parameters. The parameters chosen for these trials were *f* = 3 Hz, number of undulations = 2, and θ_max_ = 30°. The time gap between each trial was such that the flow in the tank was equal to zero before the start of the next trial. Similarly, the force measurements were also measured in five consecutive trials. In each trial the fin was actuated with *f* = 3 Hz, number of undulations = 2, and θ_max_ = 30°.

### Numerical Method

Numerical simulations of the ribbon fin were carried out using an implementation of the constraint-based immersed body (IB) method (cIB) [24,25,67–70]. The cIB involves the solution of the mass and momentum conservation equations, the Navier-Stokes equations, for the combined fluid and solid domain with a constraint force in the solid domain to impose the solid motion on the fluid. The Navier-Stokes equations for the fluid flow are solved in the entire computational domain in an Eulerian framework. The motion and kinematics of the immersed body (ribbon fin in the present work) are represented in a Lagrangian frame. Information between the Lagrangian frame of the IB and Eulerian frame of the fluid is exchanged through the discrete delta function operator [71]. Further details of the numerical technique can be found in previously published work [24,25,63]. The cIB method has been incorporated within the IBAMR software [24,25]. IBAMR is an IB method implementation with support for adaptive mesh refinement and distributed memory parallelism.

Two kinds of fin simulations were carried out: free swimming and stationary. In free swimming fin simulations, the translational degree of freedom of the swimmer being simulated is free. The swimming speed of the swimmer is determined by the solution of the numerical simulation. In the stationary fin simulations, all the translational degrees of freedom of the swimmer are locked and the swimmer is held stationary while it undulates its body as it would during free swimming. These simulations are used to compute the propulsive force generated by the swimmer’s undulations. Simulations were carried out on two sets of fins: a knifefish-sized fin (10 cm × 1 cm) and a smaller rectangular fin (2 cm × 0.4 cm). The computational cost of numerical simulations for the smaller fin is lower than that of knifefish sized fin. Because of this relatively lower computational cost, the smaller fin was used in the parametric study (Fig 4). Details of the parametric study based on the smaller fin are presented in S1 Methods Section 3. The physical properties of the fluid in the simulations were for water at 25°C: Density ρ = 0.9956×10^3^ kg/m^3^, viscosity μ = 0.89×10^−3^ N∙s/m^2^.

### Fin Kinematics

In prior work, we have approximated the shape of the traveling waves along the fins of knifefish with a traveling sinusoid [19,20,39]. The sinusoidal approximation departs from measured wave shapes in several regards [22,55], but these differences appear to have negligible impact on our results. Consequently, the kinematics of the fin undulations in the experiments and numerical simulations are described by

$$\theta =\theta_{\text{max}}\;\text{sin}2\pi (x/\lambda -ft),$$ (4)

where θ is the angular displacement of a fin ray located at an axial distance *x* from the rostral end of the fin, θ_max_ is the maximum possible angle of excursion, λ is the wavelength, and *f* is the frequency of undulation. The kinematic variables of the fin are shown in S1 Fig.

### Computing Environment

The computational resources for the numerical simulations presented in this work were provided by the Quest high performance computing facility at Northwestern University. The Quest high performance cluster is composed of 252 nodes of Intel Westmere X5650 processors with 48 GB memory/node, 68 nodes of Intel Sandybridge E2670 processors with 68 GB memory/node, and 110 Intel IvyBridge E5-2680 processors with 128 GB memory/node.

### Measurement of SW

S1 Methods Section 1 discusses the measurement of SW across the species included in Fig 1, as well as the calculation of mean amplitude.

## Supporting Information

S1 Data Data plotted in Fig 2. (XLS)

S2 Data Data plotted in Fig 3. (XLS)

S3 Data Data plotted in Fig 4. (XLS)

S4 Data Data plotted in Fig 5. (XLS)

S5 Data Data plotted in Fig 6. (XLS)

S6 Data Data plotted in S5 Fig. (XLS)

S7 Data Data plotted in S7 Fig. (XLS)

S8 Data Data plotted in S8 Fig. (XLS)

S1 Fig A schematic of the ribbon fin, which is composed of a collection fin rays that are interconnected by the fin membrane. Each fin ray (a representative ray is indicated in blue) oscillates sinusoidally around a pivot attached to the fin base with maximum angular excursion *θ*_max_, and frequency *f*. The spatial wavelength of the traveling wave along the fin is given by *λ*.
(PDF)

S2 Fig Front view and side view (corresponding to Y-Z plane and X-Z plane in Fig 7, respectively) of a ribbon fin whose height increases from *h* ^1^ for the first fin ray to *h* ^11^ for the last fin ray. The front view shows angular oscillations of ray numbers 1, 6, and 11, along with the corresponding distal amplitude and average amplitude. The maximum angle of excursion (depicted as θ as opposed to θ_max_) is constant across all the fin rays.
(PDF)

S3 Fig Measurement of fin ray length for *Amia calva*. The rays highlighted in blue are the rays for which amplitude is known. The red line is the fin base. The dotted line joining rays that are at inflexion point of the undulation is used to measure the length of fin rays which are in an undulated state. The orange line represents the scale bar, which is equal to one cm. Image courtesy of Christopher Sanford (Professor, Hofstra University).
(PDF)

S4 Fig A) Ghostbot—The robotic knifefish. B) A schematic of the experimental setup used for measuring the force generated by the undulations of the Ghostbot fin. Image taken by the authors.
(PDF)

S5 Fig Results of a parametric study of the robotic fin plotted against number of undulations in (A) to (C). (A) *L* = 32.6 cm, *f* = 4 Hz, and θ_max_ = 30°. Fin height is varied. (B) *L* = 32.6 cm, *h* = 5 cm, and *f* = 3 Hz. Maximum angle of excursion is varied. (C) *L* = 32.6 cm, *h* = 5 cm, and θ_max_ = 30°. Frequency of undulations is varied. Results of a parametric study of the simulated fin plotted against number of undulations in (D) to (F). (D) *L* = 2 cm, *f* = 1 Hz, and θ_max_ = 30°. Fin height is varied. (E) *L* = 2 cm, *h* = 0.4 cm, and *f* = 1 Hz. Maximum angle of excursion is varied. (F) *h* = 2 cm, *f* = 1 Hz, and θ_max_ = 30°. Fin length is varied. The data are available in S6 Data.
(PDF)

S6 Fig Triangular (A) and parabolic (B) fins used in numerical simulations to investigate the effect of fin morphology on the OSW. (PDF)

S7 Fig The propulsive force generated by a triangular fin and a parabolic fin plotted as a function of SW. Even when the morphology of the fin is varied, the OSW is not affected. Simulation parameters: *L* = 2 cm, *f* = 1 Hz, and θ_max_ = 30°. The data are available in S7 Data.
(PDF)

S8 Fig The swimming speed of a robotic stingray from the work of Yang et al. [27]. The robotic stingray had two triangular pectoral fins similar to a cownose ray. The swimming speed is maximum at the OSW. Experimental parameters: *L* = 30 cm, *f* = 1 Hz, and θ_max_ = 45°. The data are available in S8 Data.
(PDF)

S1 Methods Supporting methods. (PDF)

S1 TableFin ray length measurement and SW calculation details for *A*. *calva*.The fin ray numbering is from the rostral end to the caudal end of the fin. The average fin ray length (fin height) is 1.29 cm. Note that the formula for θ_max_ is written for a ray at which amplitude is given. In this example, the calculation of θ_max_ is carried out for the 10th and 36th rays (*i* = 10 and *i* = 36). Since θ_max_ is slightly different for the 10th and 36th rays, the average of the two values ($\theta_{\text{max}}^{\text{avg}}$) is used in computing the mean amplitude.(PDF)

S2 Table Size and SW of organisms studied in this work. The mean and STD of SW of the organisms investigated are 19.49 and 2.99, respectively. *Disc length in case of batoid fishes. ^†^Approximate range of body length (mantle length in case of *S*. *officinalis*). ^‡^Mean SW of forward and backward counterpropagating waves.
(PDF)

S3 Table Percentage change in the thrust force at different SWs from the force at OSW. (PDF)

S4 Table SW for five specimens of *E*. *virescens* from [40]. In this data, the fish exhibits two counterpropagating waves along its median fin. We denote the rostral wave as the head wave and the caudal wave as the tail wave.
(PDF)

S1 Video *Pseudobicoros bedfordi*, courtesy of Charlie Christie of FatFishMovies.com. (MP4)

## Abbreviations

OSW: optimal specific wavelength
SW: specific wavelength
STD: standard deviation
St: Strouhal number
