# Supplementary figures and images for: Convergent Evolution of Mechanically Optimal Locomotion in Aquatic Invertebrates and Vertebrates
Source: PLoS Biol. 2015 Apr 28;13(4):e1002123. doi: 10.1371/journal.pbio.1002123 (PMC4412495; doi:10.1371/journal.pbio.1002123)

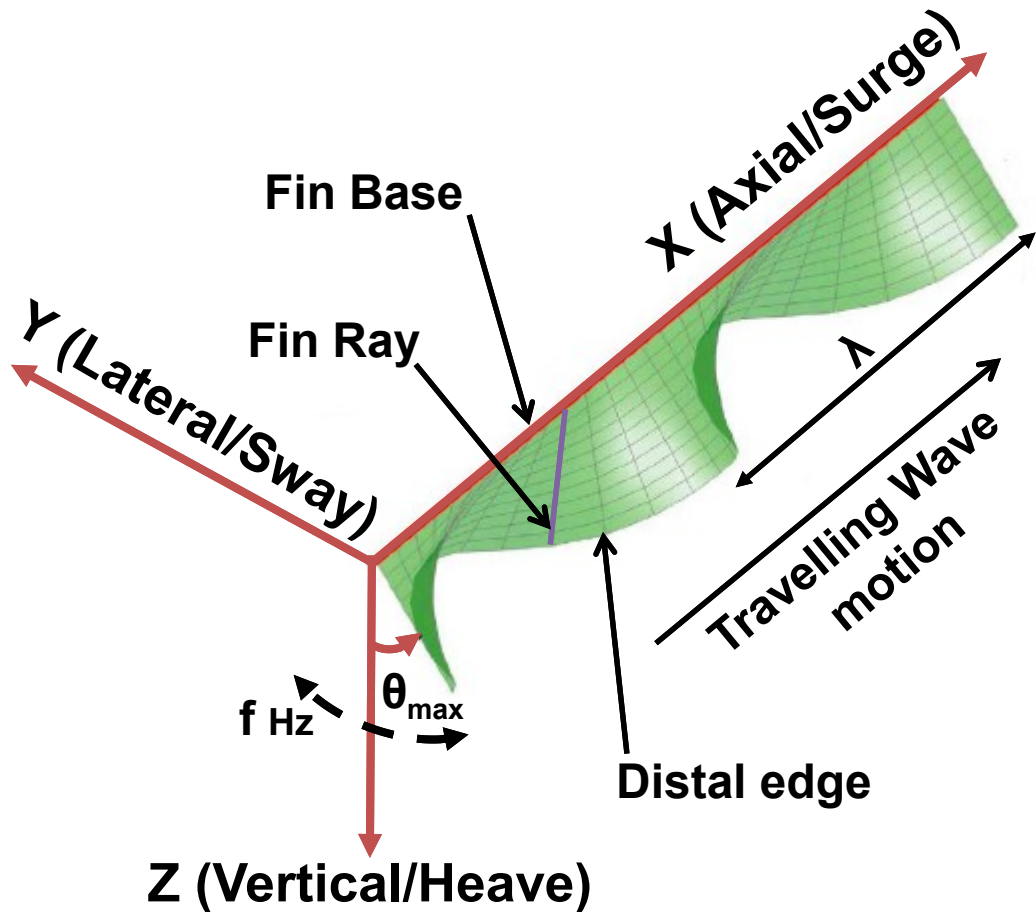

Supplement: S1 Fig — Each fin ray (a representative ray is indicated in blue) oscillates sinusoidally around a pivot attached to the fin base with maximum angular excursion θ max, and frequency f. The spatial wavelength of the traveling wave along the fin is given by λ. (PDF) [file pbio.1002123.s009.pdf]

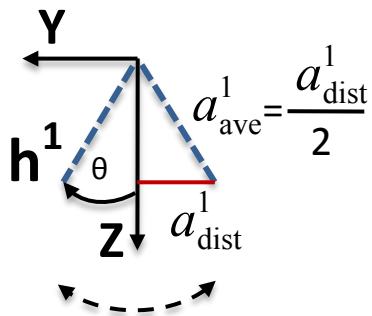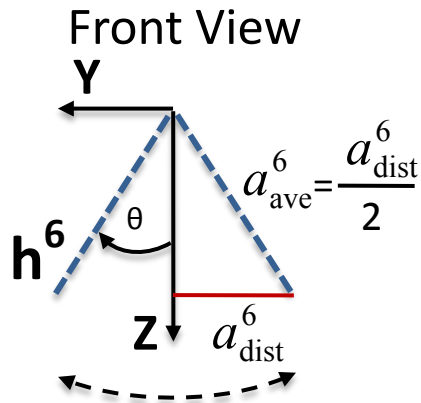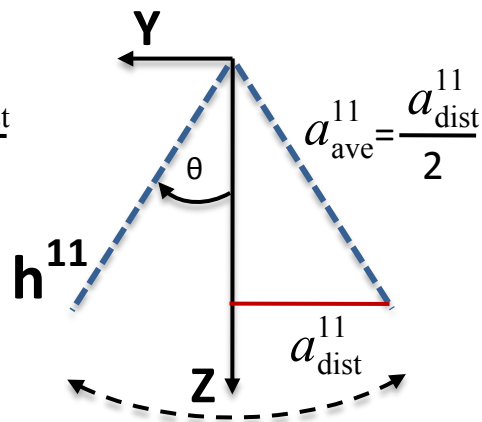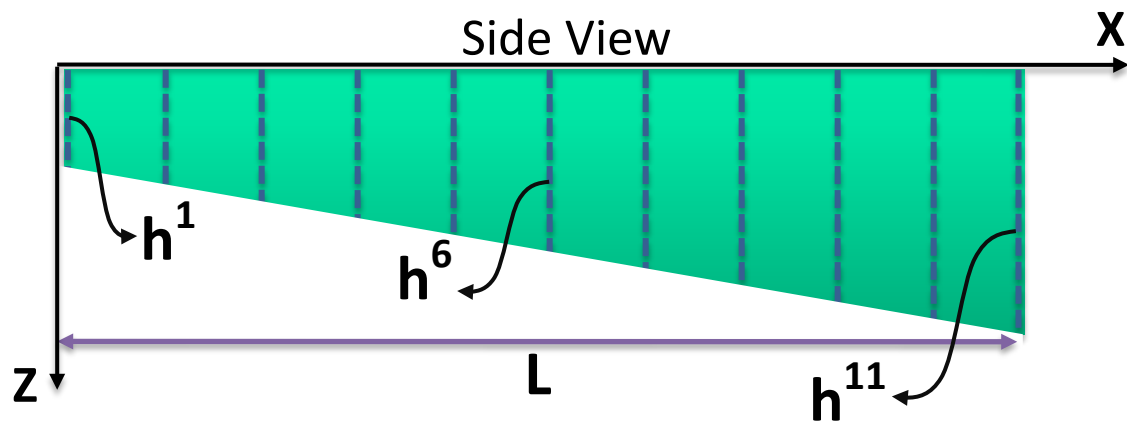

Supplement: S2 Fig — The front view shows angular oscillations of ray numbers 1, 6, and 11, along with the corresponding distal amplitude and average amplitude. The maximum angle of excursion (depicted as θ as opposed to θmax) is constant across all the fin rays. (PDF) [file pbio.1002123.s010.pdf]

1 cm

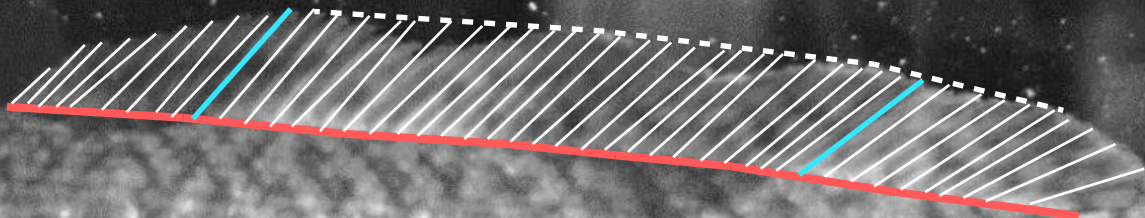

Supplement: S3 Fig — The rays highlighted in blue are the rays for which amplitude is known. The red line is the fin base. The dotted line joining rays that are at inflexion point of the undulation is used to measure the length of fin rays which are in an undulated state. The orange line represents the scale bar, which is equal to one cm. Image courtesy of Christopher Sanford (Professor, Hofstra University). (PDF) [file pbio.1002123.s011.pdf]

A

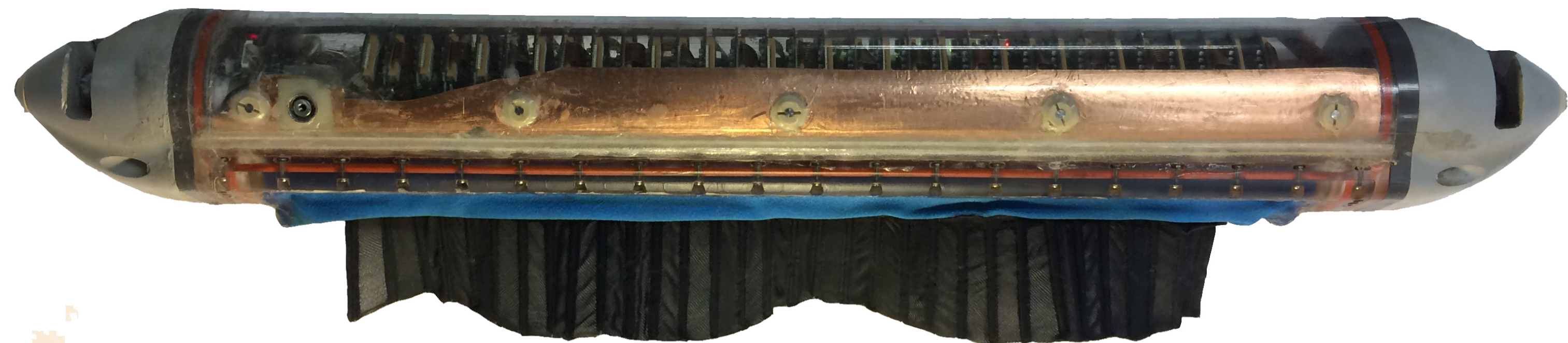

B

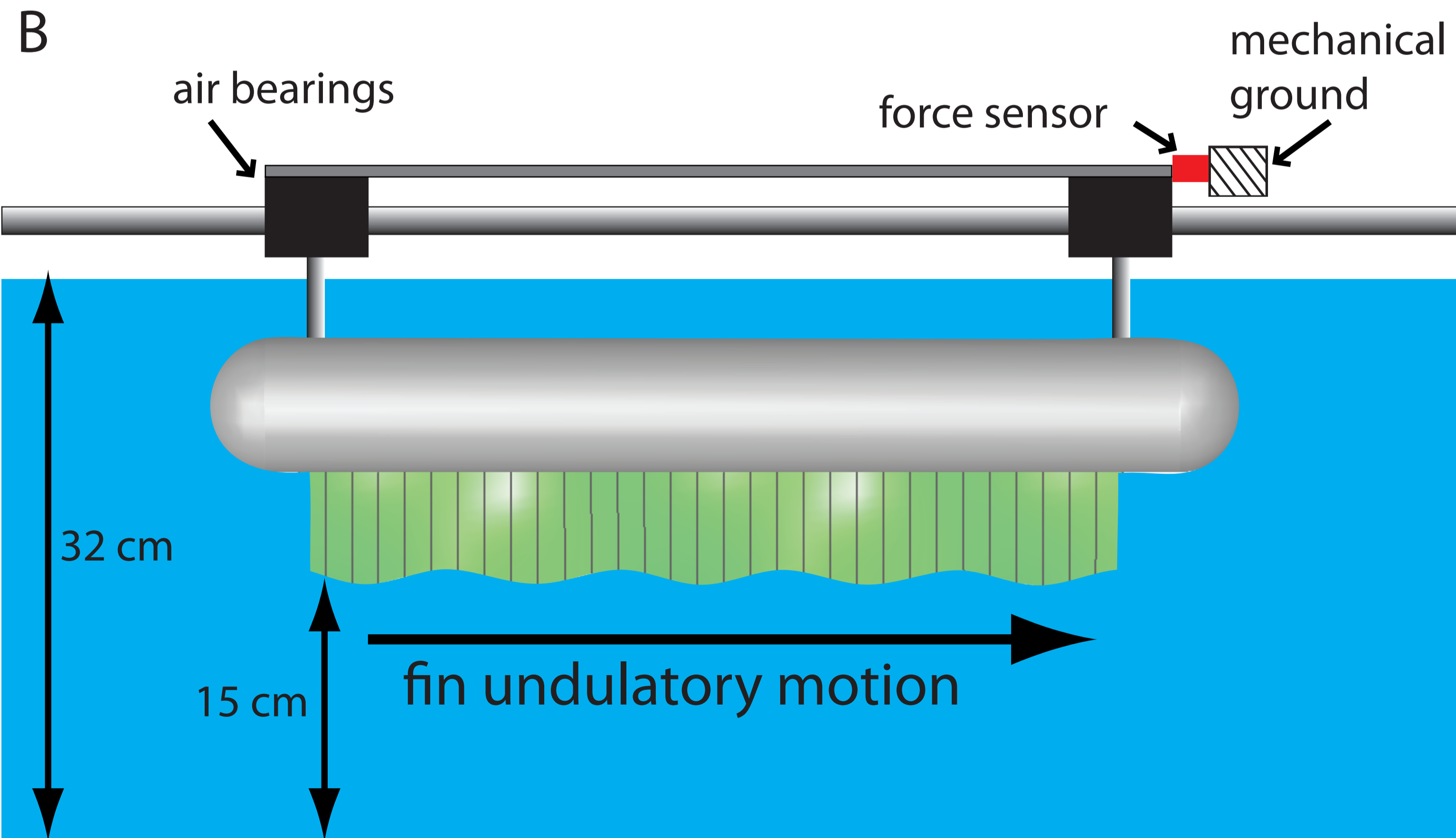

Supplement: S4 Fig — B) A schematic of the experimental setup used for measuring the force generated by the undulations of the Ghostbot fin. Image taken by the authors. (PDF) [file pbio.1002123.s012.pdf]

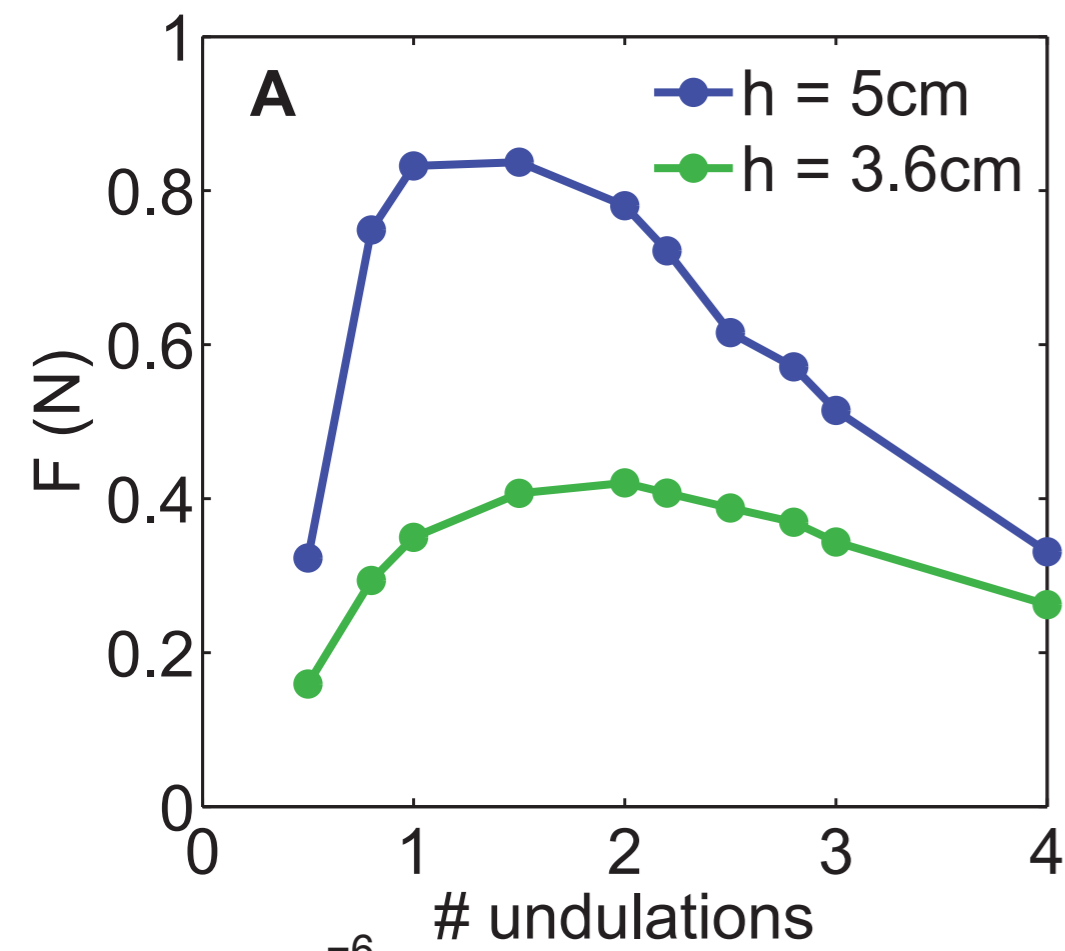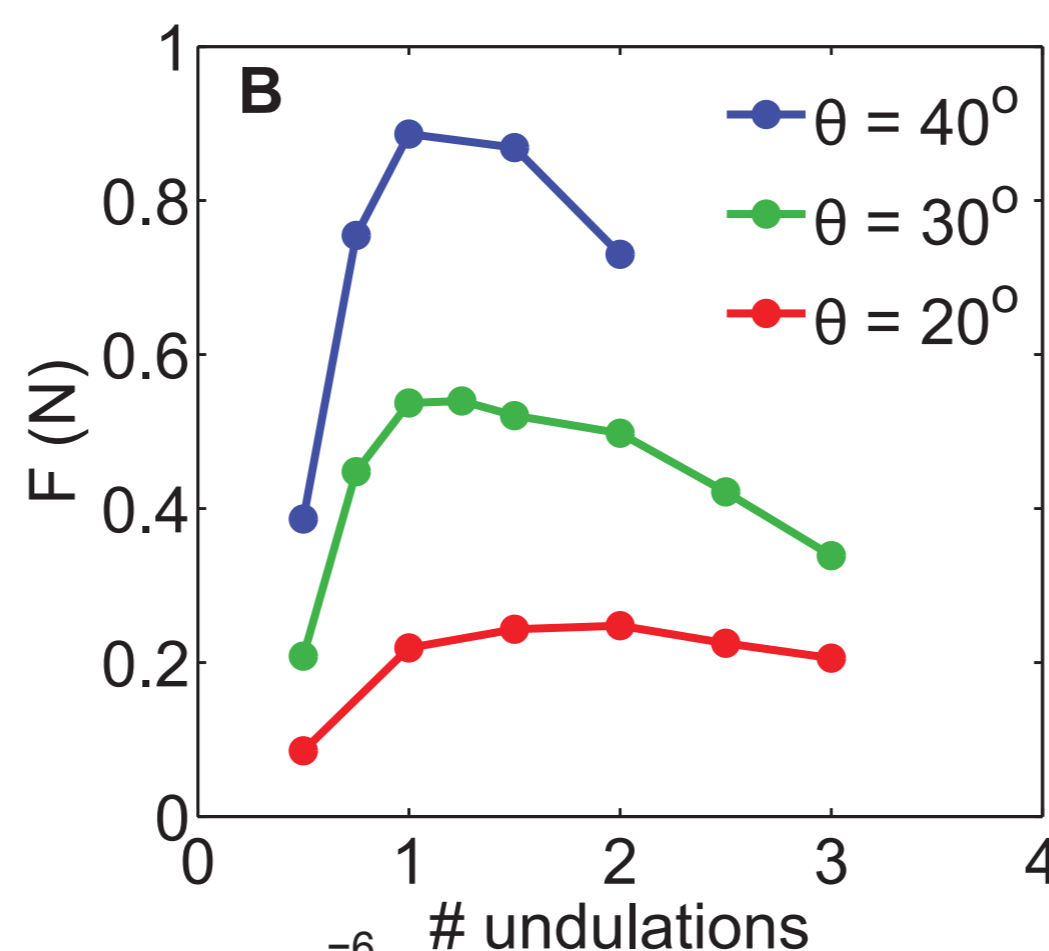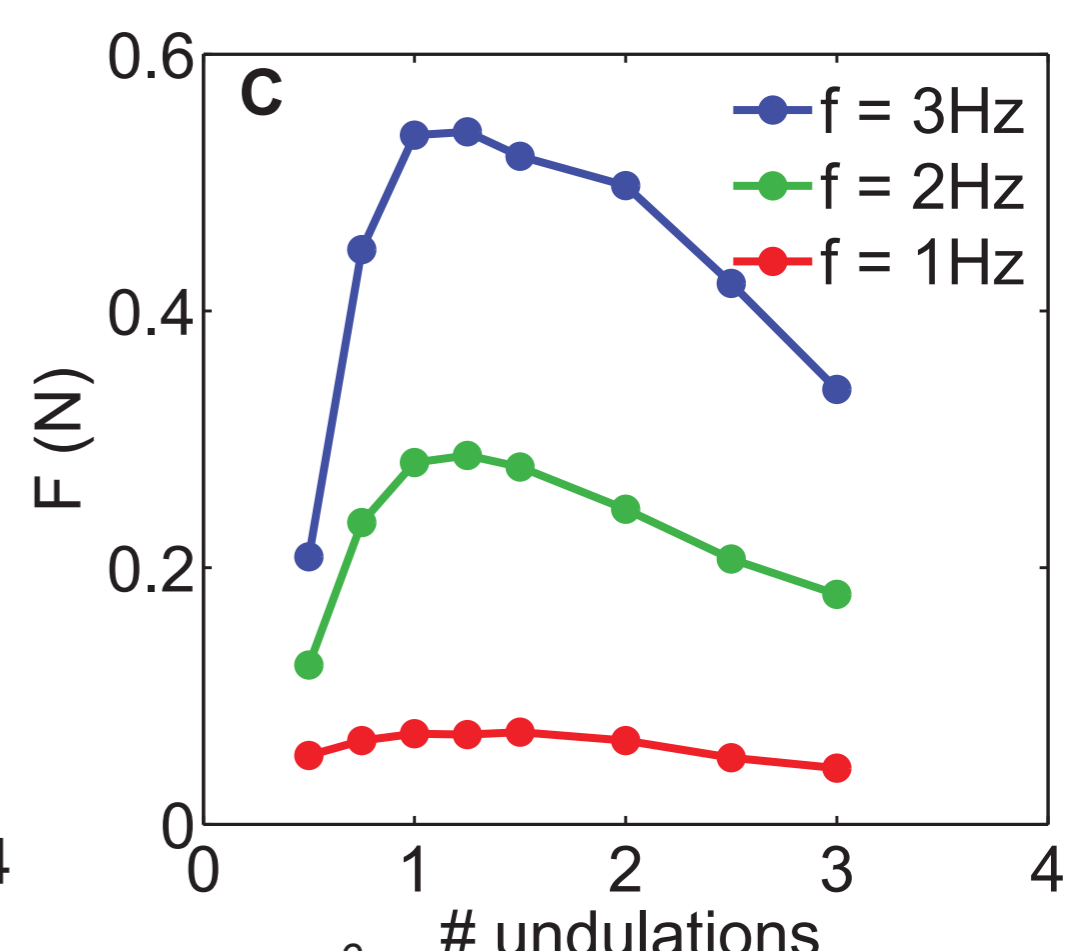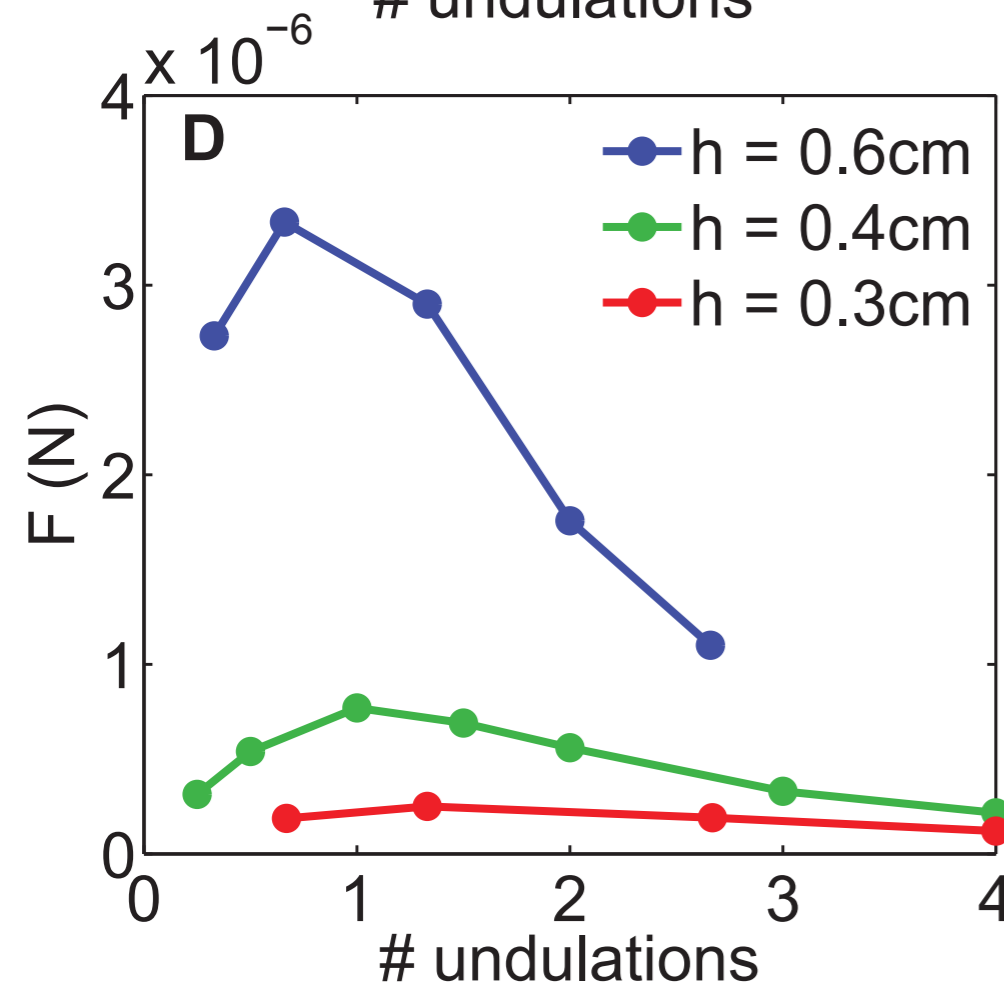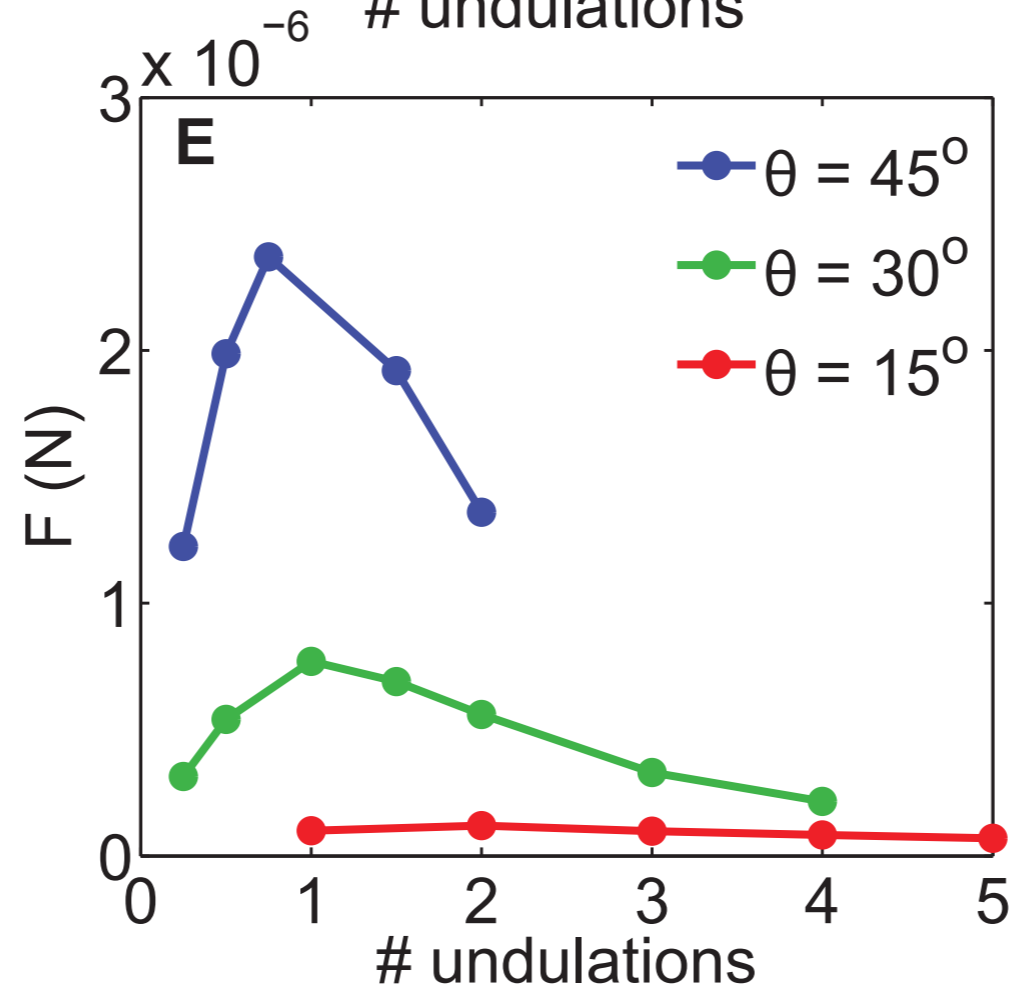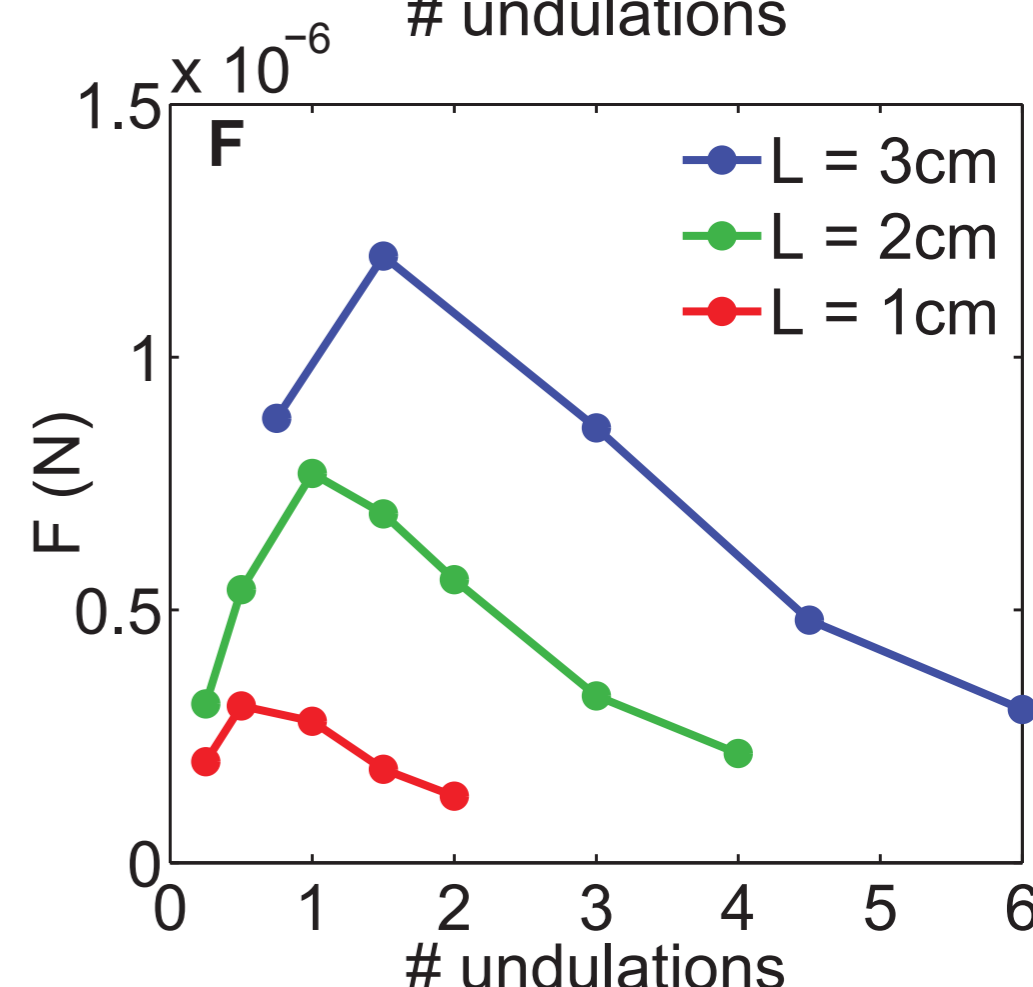

Supplement: S5 Fig — (A) L = 32.6 cm, f = 4 Hz, and θmax = 30°. Fin height is varied. (B) L = 32.6 cm, h = 5 cm, and f = 3 Hz. Maximum angle of excursion is varied. (C) L = 32.6 cm, h = 5 cm, and θmax = 30°. Frequency of undulations is varied. Results of a parametric study of the simulated fin plotted against number of undulations in (D) to (F). (D) L = 2 cm, f = 1 Hz, and θmax = 30°. Fin height is varied. (E) L = 2 cm, h = 0.4 cm, and f = 1 Hz. Maximum angle of excursion is varied. (F) h = 2 cm, f = 1 Hz, and θmax = 30°. Fin length is varied. The data are available in S6 Data. (PDF) [file pbio.1002123.s013.pdf]

**A**

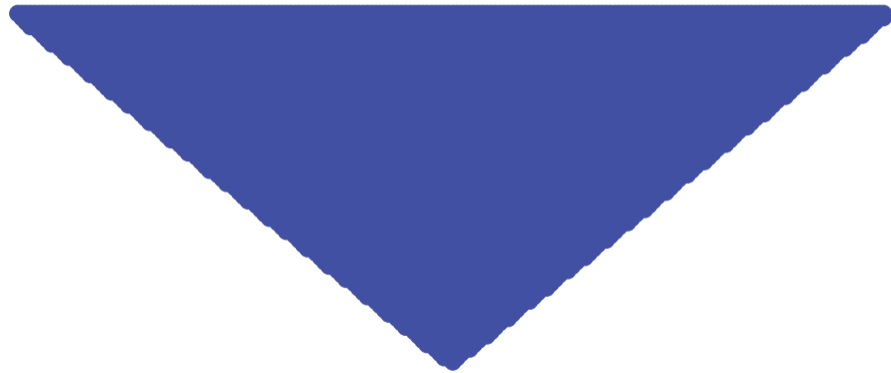

**B**

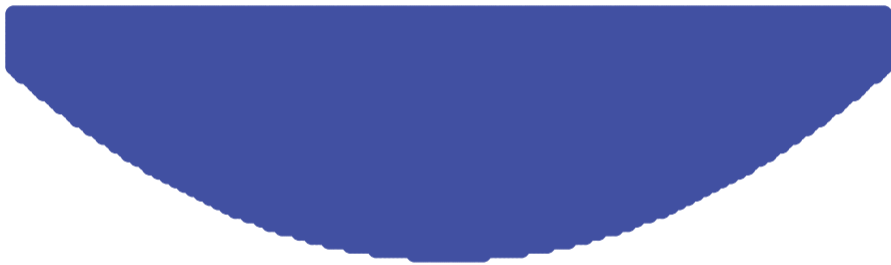

Supplement: S6 Fig — (PDF) [file pbio.1002123.s014.pdf]

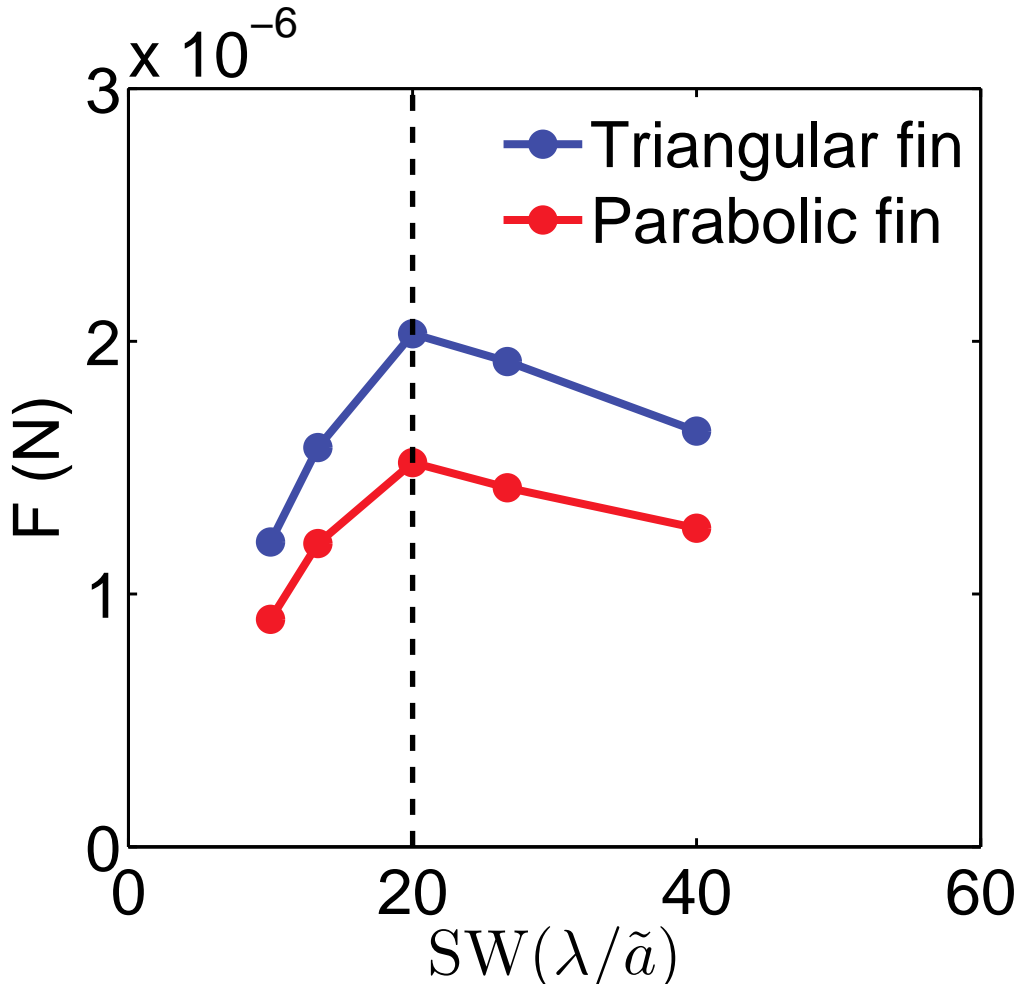

Supplement: S7 Fig — Even when the morphology of the fin is varied, the OSW is not affected. Simulation parameters: L = 2 cm, f = 1 Hz, and θmax = 30°. The data are available in S7 Data. (PDF) [file pbio.1002123.s015.pdf]

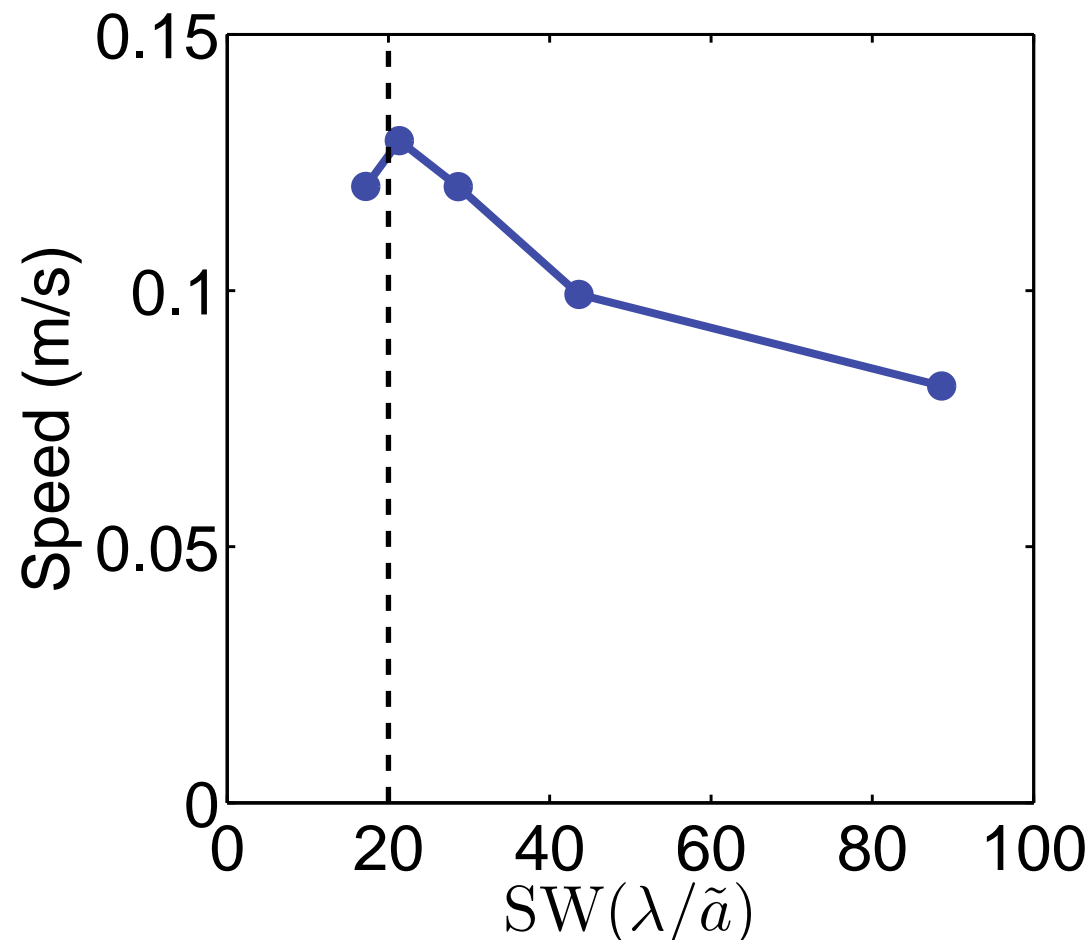

Supplement: S8 Fig — The robotic stingray had two triangular pectoral fins similar to a cownose ray. The swimming speed is maximum at the OSW. Experimental parameters: L = 30 cm, f = 1 Hz, and θmax = 45°. The data are available in S8 Data. (PDF) [file pbio.1002123.s016.pdf]
