# Supplementary material for: Convergent Evolution of Mechanically Optimal Locomotion in Aquatic Invertebrates and Vertebrates
Source: PLoS Biol. 2015 Apr 28;13(4):e1002123. doi: 10.1371/journal.pbio.1002123 (PMC4412495; doi:10.1371/journal.pbio.1002123)
Supplement: S1 Methods — (PDF) [file pbio.1002123.s017.pdf]

Supporting Information: Convergent evolution of mechanically optimal locomotion in aquatic invertebrates and vertebrates

Rahul Bale[1], Izaak D. Neveln[2], Amneet Pal Singh Bhalla[1], Malcolm A. MacIver[1,2,3,*], Neelesh A. Patankar[1,*]

1 Department of Mechanical Engineering, Northwestern University, Evanston, IL, USA

2 Department of Biomedical Engineering, Northwestern University, Evanston, IL, USA

3 Department of Neurobiology, Northwestern University, Evanston, IL, USA

∗ **E-mails: n-patankar@northwestern.edu, maciver@northwestern.edu**

1 Kinematic and morphological measurements

The specific wavelengths of the aquatic animals in Fig. 1 of the Manuscript were computed using kinematic and morphological data available in literature [3–10]. The data required for computing specific wavelength are the wavelength of undulations and the amplitude profile along the distal edge of the fin. Most organisms do not use a fixed constant wavelength to produce fin undulations. Typically there is a variation of wavelength along the fin length. For our calculations we use an averaged wavelength which is given by the ratio of fin length and number of undulations. For *Dasyatis sabina*, *Dasyatis say*, *Dasyatis americana*, *Gymnura micrura*, *Rhinoptera bonasus*, *Dasyatis violecea*, and *Raja eglanteria* the amplitude data were available at only one point along the fin [3] (for all other organisms amplitude data were available at two or more points). For these fishes, the morphological data of the fin are necessary to compute the mean amplitude. However, morphological data of all the animals we studied are not available in literature. Hence, we used size measurements of the fishes available in Rosenberger's [3] work along with specimen images of the fishes to extract fin morphology. For *Taenuira lymma* [4], *Potamotrygon orbignyi* [5], *Gymnarchus niloticus* [6, 7], *Apteronotus albifrons* [10], *Rhinecanthus aculeatus* [8], and *Amia calva* [11] both kinematic as well as morphological data were available in the literature. For *Regalecus glesne*, the video corresponding to observation 5 of the work by Benfield *et al.* [12] was used to estimate the angle of excursion, the number of undulations, and the fin height profile; the length of the oarfish reported to be 2.55 m was used in the measurement of the angle of excursion and the fin height [12].

Kinematic and morphological data for *Pseudobiceros bedfordi*, *Pseudobiceros parladis*, and *Sepia officinalis* were not available in the literature. The kinematic data, i.e., amplitude and number of undulations

on the fin during forward swimming, were measured from S1 Video, [13], and [14]. Specimen images of these three invertebrate swimmers were used to extract fin morphology; as size measurements were not available, measurements were made as a function of body length and all further calculations were carried out using these normalized measurements.

Calculation of mean amplitude $\tilde{a}$

Species for which amplitude at only one point on the fin was available, the amplitude (a_p) at that point and the distance of the point from the fin base (h_p) were used to compute the maximum angle of excursion, $\theta_{\text{max}} = \sin^{-1}\frac{a_p}{h_p}$. It is assumed that the organism moves the whole fin with this angle of excursion. It was found that in cases where amplitude profile along the whole fin was available, the maximum angle of excursion did not vary significantly along the fin length.

To demonstrate the calculation of $\tilde{a}$ we consider a fin in which the height of the fin increases from rostral end to the caudal end. As shown in S2 Fig., the height of the fin increases from h^1 to h^{11} from the first fin ray (see S1 Fig. for details of ribbon terminology) to the eleventh fin ray, respectively. With the maximum angle of excursion known from measurements as described above, length of the ith fin ray, h^i, is used to compute the distal amplitude of the ith fin ray, $a^i_{\text{dist}} = h^i \sin\theta_{\text{max}}$. As can be seen from the front view of the fin ray oscillations in S2 Fig., the amplitude of a fin ray varies from zero, at fin base, to a^i_{dist}, at the distal end. Thus, the average amplitude of a fin ray is given by $a^i_{\text{ave}} = a^i_{\text{dist}}/2$. The mean amplitude $\tilde{a}$ of the entire fin (inclusive of all the rays) is mean of the average ray amplitude given by

$$\tilde{a} = \frac{1}{N}\sum_{i=1}^{N} a^i_{\text{ave}} = \frac{1}{2N}\sum_{i=1}^{N} a^i_{\text{dist}}, \tag{1}$$

$$\tilde{a} = \frac{1}{2N}\sum_{i=1}^{N} h^i \sin\theta_{\text{max}}, \tag{2}$$

where N is the total number of rays ($N = 11$ in S2 Fig.). Species for which amplitude at multiple locations along the fin base was available, measured amplitude data from literature was directly substituted into Eq. 1 above to compute $\tilde{a}$.

As an example we demonstrate the above calculation for *Amia calva* (bowfin). The amplitudes of the 10th and 36th fin rays (from rostral end) were reported by Jagnandan and Sanford [11], which was equal to 0.91 cm at the distal end of the fin rays. Using the height of the 36th fin ray, 2.08 cm, the maximum

angle of excursion was computed to be 0.45 radians. Similarly, the angle of excursion for the 10th ray was found to be 0.48 radians. S3 Fig. shows the measurement of the fin ray length for the bowfin. Eq. 2 can be simplified as $\tilde{a} = \frac{1}{2N} \sin \theta_{\text{max}}^{\text{avg}} \sum_{i=1}^{N} h^i$ by using the average maximum angle of excursion of the 10th and 36th fin rays. $h_{\text{ave}} = \frac{1}{N} \sum_{i=1}^{N} h^i$ is the average fin ray length. Substituting this in the previous equation we have $\tilde{a} = \frac{h_{\text{ave}}}{2} \sin \theta_{\text{max}}^{\text{avg}}$. The average fin ray length (h_{ave}) for the bowfin was measured to be 1.88 cm. Substituting all these values into the previous equation, the mean fin amplitude was found to be $\tilde{a} = 0.42$ cm. The reported wavelength for bowfin is 6.46 cm. The specific wavelength, which is the ratio of wavelength to mean amplitude, is then equal to 15.68 cm. These calculations are also tabulated in S1 Table.

2 Verification of independent emergence of undulatory/oscillatory MPF swimming

The existence or the absence of undulatory/oscillatory MPF swimming was established by investigating the immediate ancestors and relatives of the groups of animals that we studied. The ancestors and relatives were checked for the presence of elongated fins and the absence of a dominant caudal fin. For example, the last common nodal point in the phylogenetic tree between the Order *Gymnotiformes* and Superorder *Acanthopterygii* is the Cohort *Clupeocephala*; all the families between the Cohort *Clupeocephala* and the Order *Gymnotiformes*, and between *Clupeocephala* and the Superorder *Acanthopterygii*, including extinct ancestors and existing relatives, were examined for the presence of undulatory/oscillatory MPF swimming. It was found that undulatory/oscillatory MPF emerged independently in *Acanthopterygii* and *Gymnotiformes*. A similar detailed examination of all the other groups of animals was carried out before concluding that undulatory/oscillatory MPF swimming evolved independently on eight separate occasions.

3 Effect of fin parameters on the optimal number of undulations

We carried out a parametric study to determine whether the optimal number of undulations for propulsive force generation is sensitive to fin shape and kinematic parameters. Fin length, fin height, frequency of

undulation, and maximum angle of excursion were the parameters whose effects were investigated. The parametric study was carried out using both experiments (using the robot and experimental setup shown in S4 Fig.) and numerical simulations.

3.1 Experimental study

The effect of variation of fin length on propulsive force generation was investigated by changing the fin length from 32.6 cm (the default fin length) to 24.5 cm and then to 16.3 cm ($\theta_{\max} = 20°$, $h = 5$ cm and $f = 3$ Hz). The fin length was reduced by cutting the fin membrane to desired lengths. The 32.6 cm fin was attached to all the fin rays, when the fin was cut to reduce the length to 24.5 cm and 16.3 cm, the fin was attached to 24 and 16 rays, respectively, counting from the rostral end. For the 24.5 cm and 16.3 cm fin length cases, the unused fin rays were behind the caudal end of the fin. An experiment was carried out to measure the force generated by the fin rays when no fin is attached to the rays, it was found that the measured force was negligible compared to the force generated when the fin membrane is present. Thus, the unused fin rays are not expected to contribute to or affect the force generated by the fin in the reduced fin length experiments. The force generated by the fin in the three cases is plotted as a function of number of undulations in Fig. 3 of the main text. As described in the main text, the number of undulations at which force generated is maximum (the optimal number of undulations) reduces with fin length. The optimal number of undulations is directly related to fin length, and is not a constant as far as the fin length is concerned.

Fins of height 5 cm and 3.37 cm ($\theta_{\max} = 30°$, $L = 32.6$ cm and $f = 4$ Hz) were used to investigate the effect of the fin height on the optimal number of undulations. Two different sets of fin rays and fin membranes were used. In both the cases fin height was equal to the ray height. The force generated by the two fins is plotted as a function of the number of undulations in S5 Fig. The optimal number of undulations increases from 1.5 to 2 as the fin height is reduced from 5 cm to 3.37 cm: the optimal number of undulations is inversely related to the fin height. To study the effect of the angle of excursion, values of 20°, 30°, and 40° were used ($L = 32.6$ cm, $h = 5$ cm and $f = 3$ Hz). In S5 Fig., it is seen that the angle of excursion has the same effect on optimal number of undulations as the fin height. The optimal number of undulations is inversely related to angle of excursion. Lastly, frequencies of 0.5 Hz, 1 Hz, 2 Hz, and 3 Hz were used to investigate the effect of frequency on the optimal number of undulations. It is evident from S5 Fig. that frequency is one parameter that has no effect on the optimal number of undulations.

3.2 Numerical study

One of the limitations of experimental analysis is the relative difficulty and cost of changing the scale of the experiment. In our case, it is not possible to investigate how the optimal number of undulations change with size (by an order of magnitude or more) of the robot's fin unless a new robot is designed at the scale in question. We resort to numerical simulations to investigate how the optimal number of undulations for propulsive force generation changes when the fin is scaled down by at least an order of magnitude. To that end, we simulated a 2 cm $\times$ 0.4 cm fin. Smaller fin size also made it possible to conduct a detailed parametric study due to reduced computational cost compared to larger fin sizes. The results of these simulations are plotted in S5 Fig. The qualitative nature of the dependence of the optimal number of undulations on height, length, and angle of excursions of the fin is the same as that in the robotic fin.

In summary, here it is demonstrated that the number of undulations is not a good dimensionless form of wavelength as far as identifying the optimal condition for propulsive force generation is concerned. We show in the main text that another dimensionless measure of wavelength called the specific wavelength is an appropriate measure to identify the optimal condition for propulsive force generation. In Fig. 4 of the Manuscript, results of the experimental investigation of the influence of various parameters on the optimal wavelength (or number of undulations) are reinterpreted in terms of the specific wavelength. In all cases, maximum propulsive force is generated very close to the same specific wavelength called the optimal specific wavelength (OSW); the optimal specific wavelength is around 20. This result is valid for simulation data as well; the maximum force generated always occurs around the optimal specific wavelength (see Fig. 4 of the Manuscript).

4 Effect of fin morphology

Through the extensive parametric study and the study of scale effects, it is now clear that the optimal specific wavelength is independent of spatio-temporal scales of the ribbon fin. Both experimental and numerical analysis are based on rectangular ribbon fins. But rarely do swimming animals in nature have perfect rectangular fins. As discussed in the main text, there is great variation in the morphology of elongated median and/or paired fins. The design of the robotic knifefish, used for the experimental analysis above, is such that it does not permit easy modifications to fin morphology. Thus, we resort

to numerical simulations to study the effect of fin morphology on optimal specific wavelength. Two fin morphologies were considered, namely, parabolic and triangular. The parabolic fin is 2 cm long and .55 cm at the deepest part of the fin (see S6 Fig.). The triangular fin is 2 cm long and 0.8 cm at the deepest part of the fin (see S6 Fig.). An angular excursion of 30° and a frequency of 1 Hz were used for both the fins. The results from the two simulations are plotted in S7 Fig. The optimal specific wavelength is unaffected by variations in fin morphologies we considered.

In experimental work on the kinematics of the pectoral fins of a robotic cownose ray, Yang *et al.* [1] investigated how the swimming speed of their robotic ray varied with the wavelength (or number of undulations). For the parameters they considered ($\theta_{\mathrm{max}} = 45°$ and $f = 1$ Hz), they found that their robot swam fastest when it undulated its pectoral fins with 0.4 waves (wavelength is 2.5 times fin length). Here we use Yang *et al.*'s [1] data to provide experimental evidence that optimal specific wavelength is not affected by fin morphology. In S8 Fig. we plot the data from Yang *et al.* as a function of specific wavelength. The swimming speed is maximum at a specific wavelength that is very close to the optimal specific wavelength (= 20), further reinforcing the robustness of the OSW.

5 Sensitivity of optimal force to specific wavelength

Biological data presented in Fig. 1 of the Manuscript and in S2 Table show that SW of swimming organisms we consider is not exactly equal to OSW, but is close to OSW. But, how close is close enough? In this section we propose a range of SW around the OSW which will define the closeness of SW to the OSW. To that end we examine the cost of deviation of SW from the OSW. The cost is quantified in terms of change in the propulsive or thrust force from the optimal force. The cost measure can then used to define a range of SW around the OSW. We define this range as the optimal range of SW (ORSW). If the SW of an organism lies within ORSW then it may said that the SW does not significantly deviate from OSW or that the SW is close to the OSW.

In S3 Table we tabulate percentage change in the thrust force from the optimal force at the OSW (= 20), as the SW is increased and decreased from the OSW. For these calculations we use the simulation and experimental data presented in Sec. 3, above. We find that that the thrust force changes by 8.43% on average when the SW is reduced by 25% from the OSW. When the SW is increased by 25% from the OSW the axial thrust reduces by 4.27% on average. Thus, even if organisms do not swim with SW equal

to OSW, the thrust force generated would differ by less than 8.43% as long as the SW is between 15 and 25, i.e., ±25% from the OSW. This range could be regarded as the ORSW.

Outside the ORSW the thrust force drops more rapidly for any additional deviation from the OSW. For example, if we consider an additional 25% deviation in SW, for a range of 10–30, the decrease in propulsive force is as much as 24.26% as seen in S3 Table.

6 Image credits for Figure 1

The following images are licensed under CC-BY: C) *Sepia officinalis* image courtesy of Hans Dappen. D) *Raja eglanteria* image courtesy of George Burgess. F) *Rhinoptera bonasus* image courtesy of Juan Aguere. J) *Taeniura lymma* image courtesy of Nicolai Johannesen. M) *Regalecus glesne* image courtesy of Sandstein. N) *Apteronotus albifrons* image courtesy of Clinton and Charles Robertson. O) *Apteronotus leptorhynchus* image courtesy of the Harvard Museum of Comparative Zoology. P) *Gymnorhamphichthys hypostomus* image courtesy of Mark Sabaj. S) *Gymnarchus niloticus* image courtesy of Masashi Kawasaki. All remaining images are public domain.

References

1. Yang SB, Qiu J, Han XY (2009) Kinematics modeling and experiments of pectoral oscillation propulsion robotic fish. Journal of Bionic engineering 6: 174–179.

2. Sefati S, Neveln ID, Roth E, Mitchell TR, Snyder JB, et al. (2013) Mutually opposing forces during locomotion can eliminate the tradeoff between maneuverability and stability. Proc Natl Acad Sci USA 110: 18798–18803.

3. Rosenberger LJ (2001) Pectoral fin locomotion in batoid fishes: undulation versus oscillation. Journal of Experimental Biology 204: 379–394.

4. Rosenberger LJ, Westneat MW (1999) Functional morphology of undulatory pectoral fin locomotion in the stingray taeniura lymma (chondrichthyes: Dasyatidae). Journal of Experimental Biology 202: 3523–3539.

5. Blevins EL, Lauder GV (2012) Rajiform locomotion: three-dimensional kinematics of the pectoral fin surface during swimming in the freshwater stingray potamotrygon orbignyi. The Journal of Experimental Biology 215: 3231–3241.

6. Fei L, Tian-Jiang H, Guang-Ming W, Lin-Cheng S (2005) Locomotion of gymnarchus niloticus: experiment and kinematics. Journal of Bionics Engineering 2: 115 –121.

7. Tian-Jiang H, Fei L, Guang-Ming W, Lin-Cheng S (2005) Morphological measurement and analysis of gymnarchus niloticus. Journal of Bionics Engineering 2: 25 –31.

8. Loofbourrow H (2009) Hydrodynamics of balistiform swimming in the Picasso triggerfish, Rhinecanthus aculeatus. Master's thesis, University of British Columbia.

9. Hu T, Wang G, Shen L, Li F (2006) Bionic inspirations of fish-like robots from rhinecanthus aculeatus. In: Mechatronics and Automation, Proceedings of the 2006 IEEE International Conference. IEEE, pp. 639–643.

10. Ruiz-Torres R, Curet OM, Lauder GV, MacIver MA (2013) Kinematics of the ribbon fin in hovering and swimming of the electric ghost knifefish. The Journal of Experimental Biology 216: 823-834.

11. Jagnandan K, Sanford CP (2013) Kinematics of ribbon-fin locomotion in the bowfin, amia calva. Journal of Experimental Zoology Part A: Ecological Genetics and Physiology 319: 569–583.

12. Benfield M, Cook S, Sharuga S, Valentine M (2013) Five in situ observations of live oarfish regalecus glesne (regalecidae) by remotely operated vehicles in the oceanic waters of the northern gulf of mexico. Journal of Fish Biology .

13. OurOceans (2008). The leopard flatworm. URL `http://youtu.be/zCH37KI_R_E`.

14. Ma S (2012). Cuttlefish swimming inside Ocean Park's Grand Aquarium, Hong Kong. URL `http://youtu.be/zjwNxbdKoeI`.

15. IBAMR: An adaptive and distributed-memory parallel implementation of the immersed boundary method. Https://github.com/IBAMR/IBAMR.

16. Blake RW (1983) Swimming in the electric eels and knifefishes. Can J Zool 61: 1432-1441.
