# Supplementary material for: Convergent Evolution of Mechanically Optimal Locomotion in Aquatic Invertebrates and Vertebrates
Source: PLoS Biol. 2015 Apr 28;13(4):e1002123. doi: 10.1371/journal.pbio.1002123 (PMC4412495; doi:10.1371/journal.pbio.1002123)
Supplement: S1 Table — The fin ray numbering is from the rostral end to the caudal end of the fin. The average fin ray length (fin height) is 1.29 cm. Note that the formula for θmax is written for a ray at which amplitude is given. In this example, the calculation of θmax is carried out for the 10th and 36th rays (i = 10 and i = 36). Since θmax is slightly different for the 10th and 36th rays, the average of the two values (θmaxavg) is used in computing the mean amplitude. (PDF) [file pbio.1002123.s018.pdf]

Ray No. (i)	Ray length (h^i) (cm)	Distal amplitude (a^i_{dist}) (cm)	$\theta_{\max} = \sin^{-1} \frac{a_p}{h_p}$ (rad)
1	0.69	-	-
2	1.08	-	-
3	1.20	-	-
4	1.33	-	-
5	1.50	-	-
6	1.58	-	-
7	1.66	-	-
8	1.68	-	-
9	1.85	-	-
10	1.96	0.91	0.48
11	1.92	-	-
12	1.92	-	-
13	1.97	-	-
14	2.00	-	-
15	1.92	-	-
16	2.03	-	-
17	2.06	-	-
18	2.08	-	-
19	2.08	-	-
20	2.05	-	-
21	2.05	-	-
22	2.03	-	-
23	2.01	-	-
24	2.00	-	-
25	2.04	-	-
26	2.03	-	-
27	2.05	-	-
28	2.01	-	-
29	2.02	-	-
30	1.98	-	-
31	2.02	-	-
32	2.04	-	-
33	2.01	-	-
34	2.06	-	-
35	2.08	-	-
36	2.08	0.91	0.45
37	2.05	-	-
38	2.08	-	-
39	2.08	-	-
40	2.05	-	-
41	2.03	-	-
42	1.98	-	-
43	1.95	-	-
44	1.87	-	-
45	1.58	-	-

Average ray length (cm) $h_{avg} = \frac{1}{N}\sum_{i=1}^{N} h^i$	Mean amplitude (cm) $\tilde{a} = \frac{h_{avg}}{2} \sin\theta^{\text{avg}}_{\max}$	Wavelength (λ) (cm)	SW ($\frac{\lambda}{\tilde{a}}$)
1.88	0.42	6.46	15.68
