# Supplementary material for: Convergent Evolution of Mechanically Optimal Locomotion in Aquatic Invertebrates and Vertebrates
Source: PLoS Biol. 2015 Apr 28;13(4):e1002123. doi: 10.1371/journal.pbio.1002123 (PMC4412495; doi:10.1371/journal.pbio.1002123)
Supplement: S2 Table — The mean and STD of SW of the organisms investigated are 19.49 and 2.99, respectively. *Disc length in case of batoid fishes. †Approximate range of body length (mantle length in case of S. officinalis). ‡Mean SW of forward and backward counterpropagating waves. (PDF) [file pbio.1002123.s019.pdf]

Organism	Body Length* (cm)	SW	Source
Dasyatis sabina	20.9	17.78	[3]
Dasyatis say	23.3	17.68	[3]
Dasyatis americana	27.7	25.09	[3]
Gymnura micrura	16	20.21	[3]
Rhinoptera bonasus	28.7	19.43	[3]
Dasyatis violecea	55	24.13	[3]
Raja eglanteria	22	19.59	[3]
Taenuira lymma	16.4	20.5	[3]
Potamotrygon orbignyi	12.8	24.03	[5]
Gymnarchus niloticus	24.5	19.02	[6, 7]
Apteronotus albifrons	14.2	18.03	[10]
Rhinecanthus aculeatus	9.79	18.1	[8]
Regalecaus glesne	255	22.47	[12]
Apteronotus leptorhynchus	15.5	19.81	[13]
Gymnotus carapo	20	22.45	[13]
Gymnorhamphichthys hypostomus	17.8	22.81	[13]
Eigenmannia virescens‡	11.59	17.7	[2]
Xenomystus nigri	15	15.05	[13]
Amia calva	30.1	15.68	[11]
Pseudobiceros bedfordi†	8 – 10	15.37	This Study
Pseudobiceros pardalis†	2 – 5	15.17	This Study
Sepia officinalis†	30 – 45	18.87	This Study

*Disc length in case of batoid fishes.
†Approximate range of body length (mantle length in case of *Sepia officinalis*).
‡Mean SW of forward and backward counterpropagating waves.
