# Supplementary material for: Convergent Evolution of Mechanically Optimal Locomotion in Aquatic Invertebrates and Vertebrates
Source: PLoS Biol. 2015 Apr 28;13(4):e1002123. doi: 10.1371/journal.pbio.1002123 (PMC4412495; doi:10.1371/journal.pbio.1002123)
Supplement: S3 Table — (PDF) [file pbio.1002123.s020.pdf]

Data type	Specific wavelength						
	10	15	20	25	30	35	40
simulation	9.68	4.84	0.00	8.87	17.74	26.61	35.48
simulation	27.27	7.79	0.00	7.47	14.94	22.40	29.87
simulation	28.33	14.17	0.00	6.69	13.37	20.06	26.75
simulation	23.52	11.76	0.00	6.21	12.59	18.98	25.37
simulation	27.27	7.79	0.00	7.47	14.94	22.40	29.87
simulation	13.00	6.39	0.00	4.03	8.50	12.97	17.43
simulation	17.54	6.48	0.00	1.44	2.87	4.31	5.74
simulation	28.74	11.89	0.00	4.34	8.68	13.02	17.36
simulation	27.27	7.79	0.00	7.47	14.94	22.40	29.87
simulation	19.65	1.76	0.00	6.64	14.18	21.71	29.24
simulation	16.19	5.92	0.00	8.74	16.48	22.28	28.09
Experiment	25.05	9.69	0.00	1.48	5.12	8.96	14.02
Experiment	25.03	5.02	0.00	0.49	7.13	16.87	29.74
Experiment	18.20	1.47	0.00	10.26	23.84	39.23	54.61
Experiment	13.58	4.75	0.00	0.52	15.25	31.29	43.52
Experiment	39.97	17.10	0.00	1.84	10.89	18.68	26.20
Experiment	25.05	9.69	0.00	1.48	5.12	8.96	14.02
Experiment	35.66	8.86	0.00	2.53	7.30	12.55	18.20
Experiment	39.97	17.10	0.00	1.84	10.89	18.68	26.20
Average	24.26	8.43	0.00	4.27	11.83	19.07	26.39
