# Supplementary material for: Convergent Evolution of Mechanically Optimal Locomotion in Aquatic Invertebrates and Vertebrates
Source: PLoS Biol. 2015 Apr 28;13(4):e1002123. doi: 10.1371/journal.pbio.1002123 (PMC4412495; doi:10.1371/journal.pbio.1002123)
Supplement: S4 Table — We denote the rostral wave as the head wave and the caudal wave as the tail wave. (PDF) [file pbio.1002123.s021.pdf]

	Fish 1		Fish 2		Fish 3		Fish 4		Fish 5	
swimming speed (cm/s)	head wave	tail wave	head wave	tail wave	head wave	tail wave	head wave	tail wave	head wave	tail wave
0.0	12.8	19.4	12.7	16.8	14.9	17.4	14.7	18.8	10.5	19.3
1.5	13.7	21.3	14.9	17.4	13.7	17.2	17.2	18.0	11.4	19.6
3.0	16.1	23.2	14.8	18.8	14.9	17.8	15.7	18.1	12.0	19.7
4.5	15.9	22.1	14.3	19.8	16.5	18.8	16.0	20.8	12.6	23.0
6.0	17.6	20.9	14.9	19.6	16.6	18.6	15.9	19.3	12.9	23.6
7.5	16.8	20.7	14.0	20.7	16.7	20.3	15.4	21.1	13.3	24.6
9.0	16.4	21.0	15.8	20.5	16.7	18.8	15.1	19.3	13.7	23.7
10.5	17.0	20.9	14.9	20.2	15.1	20.0	15.3	22.7	14.4	25.5
12.0	13.1	33.3	14.2	22.0	15.2	22.9	15.7	n/a	13.5	26.0
